# PDAC-derived exosomes enrich the microenvironment in MDSCs in a *SMAD4*-dependent manner through a new calcium related axis

**DOI:** 10.18632/oncotarget.20863

**Published:** 2017-09-13

**Authors:** Daniela Basso, Elisa Gnatta, Andrea Padoan, Paola Fogar, Sara Furlanello, Ada Aita, Dania Bozzato, Carlo-Federico Zambon, Giorgio Arrigoni, Chiara Frasson, Cinzia Franchin, Stefania Moz, Thomas Brefort, Thomas Laufer, Filippo Navaglia, Sergio Pedrazzoli, Giuseppe Basso, Mario Plebani

**Affiliations:** ^1^ Department of Medicine – DIMED, University of Padova, Padova, Italy; ^2^ Department of Biomedical Sciences, University of Padova, Padova, Italy; ^3^ Proteomic Center, University of Padova, Padova, Italy; ^4^ Department of Woman and Child Health, Oncohematology Laboratory, University of Padova, Padova, Italy; ^5^ Eurofins Medigenomix GmbH, Ebersberg, Germany; ^6^ Comprehensive Biomarker Center GmbH (Recently re-named to Hummingbird Diagnostics GmbH), Heidelberg, Germany; ^7^ Association Wirsung Onlus, Padova, Italy

**Keywords:** pancreatic cancer, exosomes, myeloid derived suppressor cells, calcium, SMAD4

## Abstract

Tumor genetics and escape from immune surveillance concur in the poor prognosis of PDAC. In this study an experimental model was set up to verify whether *SMAD4*, deleted in about 55% PDAC and associated with poor prognosis, is involved in determining immunosuppression through Exosomes (Exo). Potential mechanisms and mediators underlying *SMAD4*-dependent immunosuppression were evaluated by studying intracellular calcium (Fluo-4), Exo-miRNAs (microarray) and Exo-proteins (SILAC). Two PDAC cell lines expressing (BxPC3-*SMAD4*+) or not-expressing (BxPC3) *SMAD4* were used to prepare Exo-enriched conditioned media, employed in experiments with blood donors PBMCs. Exo expanded myeloid derived suppressor cells (gMDSC and mMDSC, flow cytometry) and altered intracellular calcium fluxes in an *SMAD4* dependent manner. BxPC3-*SMAD4*+, but mainly BxPC3 Exo, increased calcium fluxes of PBMCs (*p* = 0.007) and this increased intracellular calcium trafficking characterized mMDSCs. The analysis of de-regulated Exo-miRNAs and transfection experiments revealed hsa-miR-494-3p and has-miR-1260a as potential mediators of *SMAD4-*associated de-regulated calcium fluxes. Eleven main biological processes were identified by the analysis of *SMAD4*-associated de-regulated Exo-proteins, including translation, cell adhesion, cell signaling and glycolysis. A reverse Warburg effect was observed by treating PBMCs with PDAC-derived Exo: BxPC3 Exo induced a higher glucose consumption and lactate production than BxPC3-*SMAD4*+ Exo. Conclusion: PDAC-derived Exo from cells with*,* but mainly from those without *SMAD4* expression, create an immunosuppressive myeloid cell background by increasing calcium fluxes and glycolysis through the transfer of *SMAD4*-related differentially expressed miRNAs and proteins.

## INTRODUCTION

Pancreatic ductal adenocarcinoma (PDAC) is still one of the most lethal forms of cancer worldwide despite the accumulating knowledge gained on its biology and genetics [1-3]. Recent studies exploring the whole genome and exome sequences revealed that the genetic landscape of PDAC is much more complex than previously believed, but they also confirmed that activating mutations of *KRAS* and inactivating mutations in the tumor suppressor genes *p53*, *CDKN2A* and *SMAD4* characterize this tumor type [4, 5]. The loss of *SMAD4*, an almost unique event in gastrointestinal malignancies (e.g. colorectal adenocarcinoma), which occurs in about 50% of PDAC [2], appears to be of particular interest. PDAC patients carrying *SMAD4* loss have a worse prognosis [6, 7], and this event, *in vitro,* favors cell proliferation and migration, while antagonizing cell senescence [8]. It is widely believed that the molecular basis linking *SMAD4* loss with increased tumor growth and aggressiveness depends on the absence of Smad4 protein, which mediates the effects of the transforming growth factor (TGF)-β and bone morphogenetic protein, which inhibit cell proliferation and migration, and trigger apoptosis [9]. Yet this underlying mechanism cannot alone explain all PDAC features associated with *SMAD4* loss, as recently demonstrated by Whittle et al. [10], who found that heterozygous *SMAD4* mutation is associated with increased cellular proliferation but attenuates the metastatic potential of PDAC, while the complete loss of SMAD4 restores metastatic competency by regulating the expression of the transcription factor Runx3. Moreover, *SMAD4* loss might underlie a more aggressive tumor pattern not only because tumor cells acquire a pro-proliferative and pro-metastatic phenotype, but also because it might create a favorable soil for tumor growth and metastasis by conditioning the surrounding stroma [11, 12].

The PDAC microenvironment, characterized by a dense desmoplastic reaction driven mainly by pancreatic stellate cells and pancreatic cancer associated fibroblasts, include immune cells, which have an imbalance toward an immunosuppressive and protumorigenic phenotype [13, 14]. Immunosuppressive T_reg_ lymphocytes, M2 polarized tumor associated macrophages (TAM) and myeloid derived suppressor cells (MDSCs) prevail over immune effector CD8^+^ T cells, dendritic cells (DCs) and M1 polarized TAM in the tumor microenvironment, in blood and in lymphoid organs in both PDAC animal models and humans [15-23]. The immune imbalance in PDAC is an evolving phenomenon, starting in the early phases of carcinogenesis (i.e. in PanINs), a specific chronology of distinct immune cells subsets derangement paralleling disease progression [24]. While lymphocyte lineage alterations with T_reg_ accumulation appear to occur early, alterations in myeloid lineage with MDSCs accumulation appear to occur later in PDAC [20]. Overall, PDAC-associated immune cell alterations concur in masking tumor cells from immune detection, thus favoring tumor progression. This observation is borne out by clinical data demonstrating the negative prognostic value of immunosuppressive cellular accumulation, and experimental research is now being conducted to establish whether therapeutic benefit might be gained by targeting immunosuppressive cells [14, 20].

Immune cell alterations at the tumor site, and in nearby and distant organs, appear to depend on tumor-derived and immunomodulatory molecules, including GM-CSF, S100A8/A9 heterocomplex and chemokines, which target the Ras/MAPK, Jak/Stat, PI3K and TGFβ pathways [14, 25]. Soluble mediators, however, are not the only means of ‘communication’ between tumor cells and the surrounding stromal cells, extracellular micro vesicles also being involved [26]. Exosomes (Exo), defined as micro vesicles with a diameter ranging from 30 to 150 nm, are released by cells and can transfer from one cell to another a rich cargo of molecular messengers, including miRNAs, proteins, lipids and DNA, which remains highly stable within the Exo shuttle [27]. The role of Exo has been highlighted in carcinogenesis, metastases, drug resistance and immunosuppression [28-31]. Among the mechanisms activated by cancer derived Exo in the specific immune cellular setting, Stat3 and NF-κB activation has been suggested to occur in a TLR-dependent fashion due to Hsp70, miR- 203 or as yet unidentified proteins [32, 33].

When investigating the immune cell response to cancer, the role of calcium signaling should be taken into account, since it plays a relevant role in the elaboration of the adaptive immune response thought to be crucial to cancer control [34, 35]. It remains to be elucidated whether and, if so, how variations in the mutational landscape of PDAC, with a special focus on *SMAD4* deletion, accelerates immunosuppression; this would lead to a greater understanding leading to new therapeutic targeting. In the present study we demonstrate that PDAC derived Exo imbalance immature myeloid cells subsets, enhancing MDSCs while lowering DCs, by altering intracellular calcium fluxes, these effects being magnified in cases of *SMAD4* loss. The characterization of miRNAs, and the protein cargo of PDAC derived Exo, allowed us to discover *SMAD4*-associated de-regulated miRNAs and proteins, and to identify, among the more extensively de-regulated miRNA, hsa-miR-494-3p and hsa-miR-1260a, and among proteins, VDAC1 and spectrin beta, all involved in calcium balance. Finally, *SMAD4* loss determined enrichment in the Exo protein cargo of glycolytic enzymes, these Exo being able to enhance myeloid cell glycolysis.

## RESULTS

### PDAC-derived Exo change the balance between MDSCs and DCs

To ascertain whether pancreatic cancer cells modify the equilibrium between immunosuppressive and immune effector cells in an *SMAD*4-dependent manner and whether any imbalance is mediated by soluble factors or Exo, PBMCs were cultured for four days in complete, Exo enriched and Exo free non conditioned (NC) and conditioned media (CM) obtained from BxPC3 and BxPC3-*SMAD*4+ cells. T cells and immature myeloid cells subsets were analyzed by flow cytometry and data from eight donors were collected. Only complete CM obtained from BxPC3-*SMAD*4+ cells caused a mild but significant expansion of CD4^+^CD25^+^ (Repeated measures ANOVA: F = 10.77, *p* = 0.003), while reducing CD8^+^ T cells (F = 8.348, *p* = 0.007) (Supplementary Figure 1).

**Figure 1:**
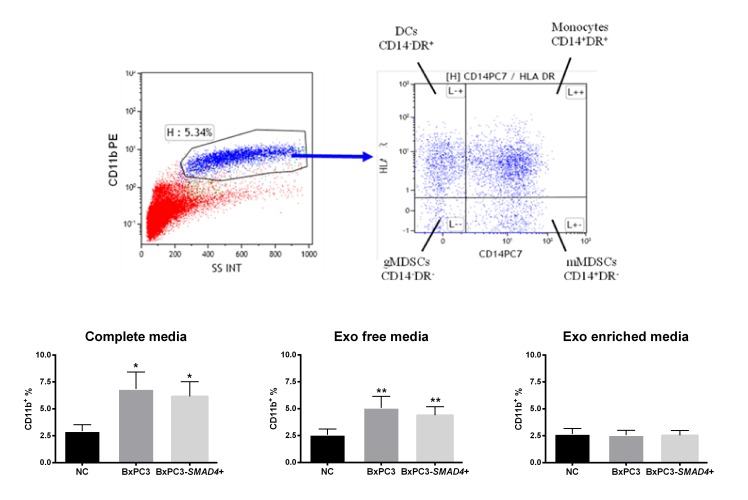
BxPC3 and BxPC3-*SMAD4*+ CM expand CD11b^+^ immature myeloid cells Upper panels: Representative CD11b gating in flow cytometry (upper left panel) and subsequent analysis of CD11b^+^ cells on the basis of CD14 and HLA-DR expression (upper right panel). DCs: Dendritic cells (CD11b^+^CD14^-^HLA-DR^+^); gMDSCs (CD11b^+^CD14^-^HLA-DR^-^) and mMDSCs (CD11b^+^CD14^+^HLA-DR^-^): granulocytic and monocytic myeloid derived suppressor cells; Monocytes (CD11b^+^CD14^+^HLA-DR^+^). Bottom panels: CD11b^+^ immature myeloid cells counted after incubating PBMCs for four days with complete, Exo free and Exo enriched BxPC3 (dark grey columns) or BxPC3-*SMAD4*+ (light grey columns) conditioned media. PBMCs were always run in parallel in complete, Exo free and Exo enriched non-conditioned medium (NC, black columns). All data are given as average values ± SEM obtained for 8 donors * = *p*<0.05 and ** = *p*<0.01 with respect to NC (Tukey’s multiple comparisons test).

Within the CD11b expressing immature myeloid cells, four subsets were identified on the basis of the expression of CD14 and HLA-DR markers: monocytes (CD14^+^DR^+^), DCs (CD14^-^DR^+^), granulocytic (gMDSCs, CD14^-^DR^-^) and monocytic (mMDSCs, CD14^+^DR^-^) MDSCs. Figure 1 shows a typical example of CD11b gating and the subsequent analysis of CD11b^+^cells on the basis of CD14 and HLA-DR expression (upper panels). Complete and Exo free CM obtained from both BxPC3 and BxPC3-*SMAD*4+ cells induced a significant increase of CD11b^+^ immature myeloid cells (Repeated measures ANOVA: F = 11.190, *p* = 0.007 and F = 15.540, *p* = 0.001 respectively), while Exo enriched fraction did not (F = 0.102, *p* = 0.793) (Figure 1, bottom panels). Cellular apoptosis (Annexin V) was not affected by any BxPC3 and BxPC3-*SMAD*4+ CM (Supplementary Table 1).

**Figure 2:**
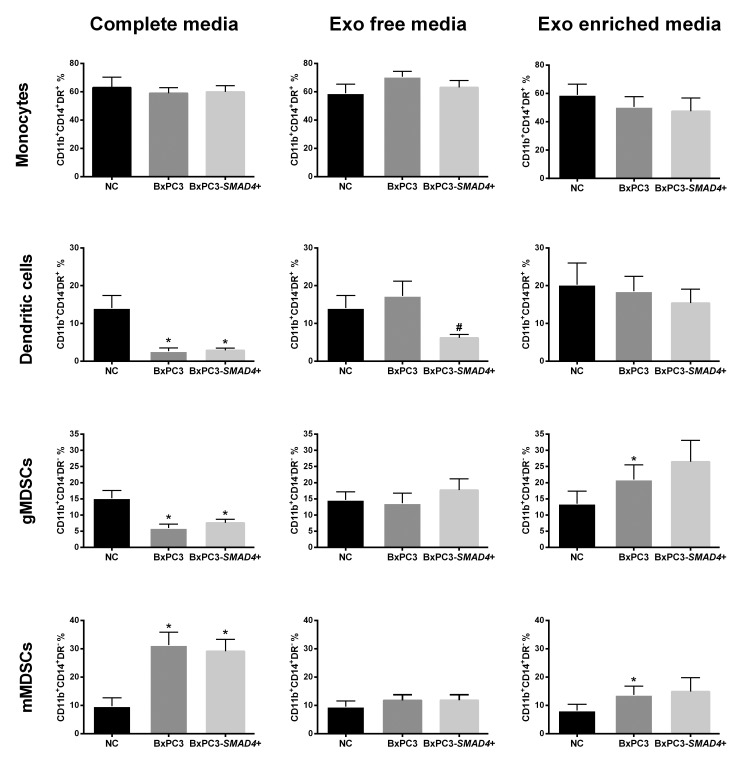
BxPC3 derived Exo expand myeloid derived suppressor cells Monocytes, dendritic cells, granulocytic and monocytic derived suppressor cells (gMDSCs and mMDSCs) were counted after PBMCs were incubated for four days with complete, Exo free and Exo enriched BxPC3 (dark grey columns) or BxPC3-*SMAD4*+ (light grey columns) conditioned media (CM). PBMCs were always run in parallel in complete, Exo free and Exo enriched non-conditioned medium (NC, black columns). All data are given as the average values ± SEM obtained from eight donors * = *p*<0.05 with respect to NC and # = *p*<0.05 with respect to NC and BxPC3 (Tukey’s multiple comparisons test).

Figure 2 illustrates the distribution of the four myeloid cells subsets obtained in the above-described experimental conditions. Complete CM obtained from both BxPC3-*SMAD*4+ and BxPC3 cells caused a significant increase in the percentage of mMDSCs (Repeated measures ANOVA: F = 12.50, *p* = 0.006) while reducing both DCs (F = 12.82, *p* = 0.009) and gMDSCs (F = 10.590, *p* = 0.013). Exo enriched media enhanced the percentage of mMDSCs (F = 3.749, *p* = 0.059) but also gMDSCs (F = 6.027, *p* = 0.034) in a *SMAD4* independent manner, while Exo free media reduced the DCs population in the presence of *SMAD4* (F = 7.711, *p* = 0.014). The CD11b^+^CD14^-^DR^+^ subset was confirmed to represent the DCs subpopulation in a separate series of two independent flow cytometry experiments with the CD80 and CD86 markers, which expression was not altered by BxPC3 or BxPC3-*SMAD4*+ CM (Supplementary Figure 2). Macrophages, classified as CD11b^+^CD16^+^ cells, were mainly allocated within the monocytes subset and in part in the mMDSCs subset, and they were not affected by treatment with cancer conditioned complete, Exo enriched and Exo free media (Supplementary Table 2).

The data obtained indicate that PDAC cells induce the expansion of CD11b^+^ immature myeloid cells in a *SMAD*4-independent manner through the release of soluble mediators. Within this expanded population of myeloid cells, PDAC through off balance between immature myeloid cells subsets, lowering DCs and gMDSCs, while enhancing mMDSCs, whether in the absence or presence of *SMAD*4 gene expression. Exo from BxPC3 appear to mediate the expansion of any immunosuppressive cell subtype.

### PDAC lowers TNF release by PBMCs in a *SMAD4*-dependent manner

IL-1β, IL-4, IL-6, IL-10, TGF-β1 and TNF were assayed in PBMCs’ supernatants before and after four days in NC and in CM. IL-1β, IL-4, IL-6, IL10 did not vary and levels were below the lower limit of detection in all conditions. The levels of TGF-β1 measured in PBMCs supernatants collected after four days of culture did not significantly vary between NC (5,18±0,46 μg/L, mean±SEM from 4 replicated experiments), BxPC3 CM (5,25±0,74 μg/L) and BxPC3-*SMAD4*+ CM (4,89±0,40 μg/L) (One-way Anova: F = 0.465, *p* = 0.556). With respect to basal levels, a significant increase of TNF was found in NC PBMCs after four days of culture (from 4.81±0.16 pg/mL to 265.4±94.9 pg/mL, mean±SEM from 19 donors; Student’s t test for paired data: t = 2.755, *p* = 0.013). The levels of TNF measured in PBMCs supernatants after four days in BxPC3 and BxPC3-*SMAD*4+ CM were expressed as percentage with respect to the corresponding TNF levels of NC PBMCs considering any donor of this series of 19 donors. The results are shown in Supplementary Table 3, which also reports the results obtained in a subset of 12/19 donors, for which parallel experiments were performed using Exo enriched NC and CM. With respect to NC media, the release of TNF by PBMCs was reduced by BxPC3-*SMAD4*+ CM, but mainly by BxPC3 CM. Exo enriched media did not reproduce the inhibitory effect observed with the employment of complete media.

### PDAC-derived Exo increase intracellular calcium fluxes in a *SMAD4* dependent manner

**Figure 3:**
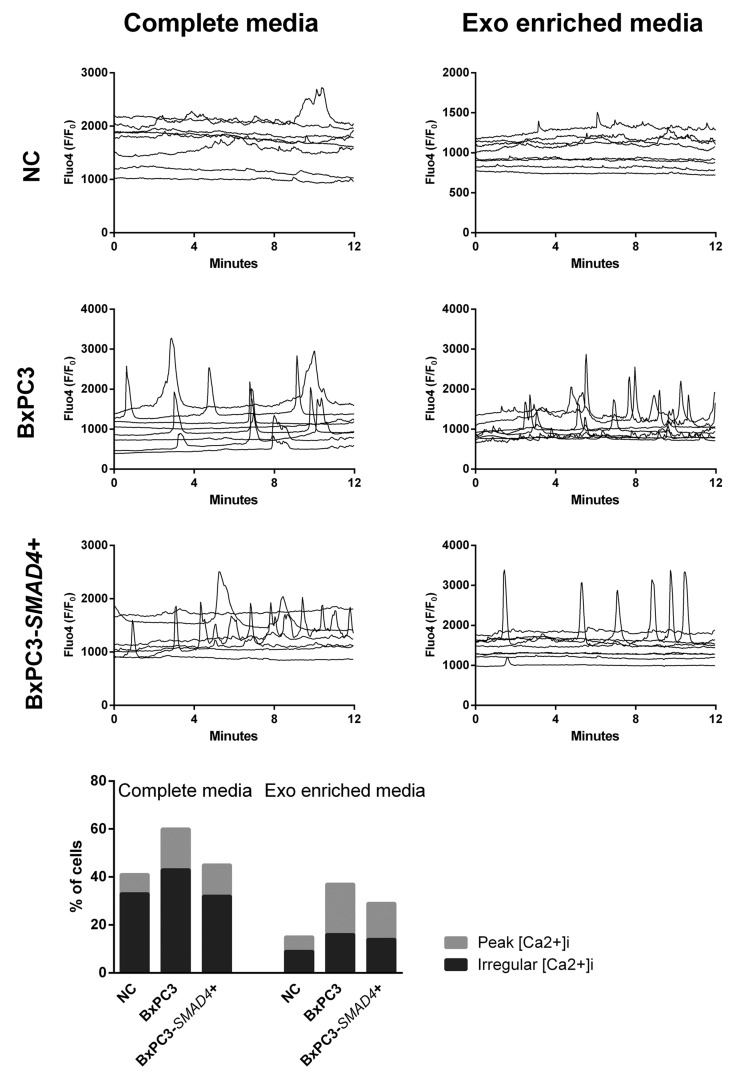
Intracellular calcium fluxes ([Ca^2+^]_i_ ) of PBMCs The variations in [Ca^2+^]_i_ after incubation for 72 hours in complete or Exo enriched non conditioned (NC) or BxPC3 and BxPC3-*SMAD4*+ conditioned media are shown as Fluo4 F/F_0_ ratio. Each graph represents one experiment and each line [Ca^2+^]_i_ of one cell. The bottom graph shows the percentages of PBMCs that peak (clear grey bars) or have an irregular [Ca^2+^]_i_ (dark grey bars) after 72 hours’ incubation in complete or Exo enriched NC or conditioned media. Totals of 162, 129 and 164 cell data were obtained from 12 independent experiments for the entire NC , BxPC3 CM and BxPC3-*SMAD*4+ CM, respectively. Totals of 108, 130 and 126 cell data were obtained from 9 independent experiments for Exo-enriched NC , BxPC3 CM and BxPC3-*SMAD*4+ CM, respectively.

With the aim to verify whether Ca^2+^ ions were involved in the observed PDAC dependent changes in myeloid cell subsets and TNF secretion, [Ca^2+^]_i_ fluxes were studied in PBMCs treated in the same conditions described above and Figure 3 shows the results of representative experiments for any studied condition each reporting results from 8 cells. Minimal and irregular variations in [Ca^2+^]_i_ were observed in NC complete and Exo-enriched media (Figure 3, upper panels), while an increased frequency of [Ca^2+^]_i_ was observed in PBMCs cultured in BxPC3-*SMAD*4+, but mainly in BxPC3 complete CM, the results being reproduced by Exo-enriched CM. To compare the results, we classified variations in [Ca^2+^]_i_ as absent or present and, in this latter case, as regular peaks or irregular oscillations. In each experiment the number of cells belonging to the first (stable [Ca^2+^]_i_), second (peak [Ca^2+^]_i_) or third group (irregular [Ca^2+^]_i_) were counted. BxPC3-*SMAD*4+, but mainly BxPC3 complete CM caused a significant increase in the number of cells with a peak or irregular behavior of [Ca^2+^]_i_ (X^2^ = 13.480, *p* = 0.009) and this finding was confirmed when cells were incubated with Exo-enriched CM (X^2^ = 14.212, *p* = 0.007). In the bottom panel of Figure 3 the percentages of PBMCs showing an irregular or a peak [Ca^2+^]_i_ pattern after they have been incubated in complete or Exo enriched NC or pancreatic cancer CM are shown.

### DCs do not pulse whereas regular intracellular calcium fluxes characterize mMDSCs

**Figure 4:**
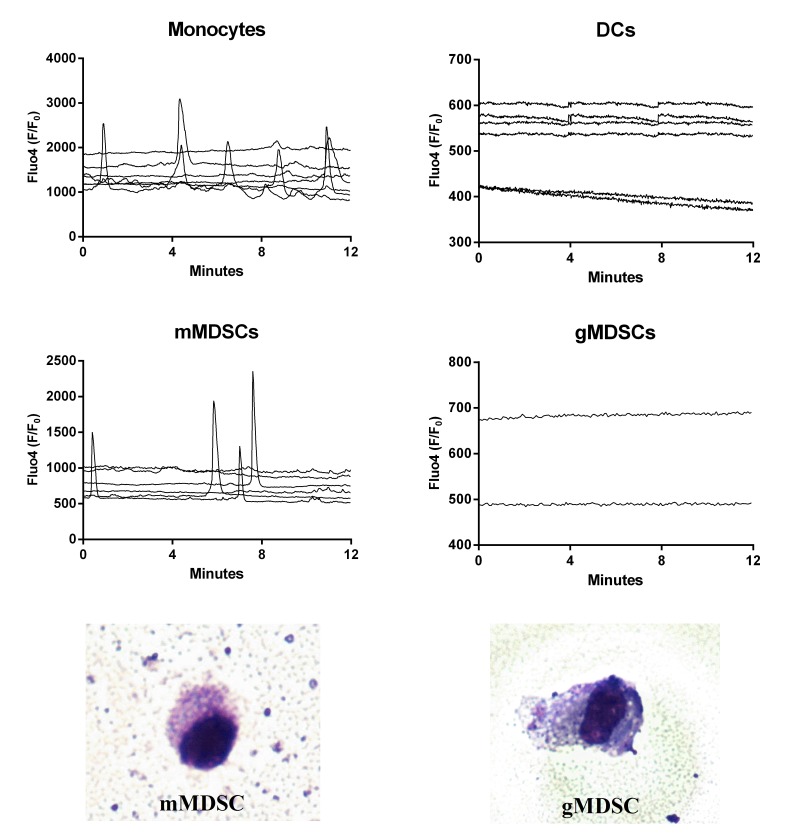
Intracellular calcium fluxes ([Ca^2+^]i) of FACS sorted immature myeloid cell subsets The variations in [Ca^2+^]_i_ of each cellular subset after sorting and incubation for 48 hours in non conditioned media are shown as Fluo4 F/F_0_ ratio. Each graph represents one experiment and each line, the [Ca^2+^]_i_ of one cell. The bottom images show FACS sorted gMDSC cell (right) and mMDSC cell (left) at light microscopy (50×) after May-Grünwald-Giemsa staining. The percentage of Annexin V+ cells (Flow cytometry) after 18 hours from sorting was 79% among monocytes, 69% among DCs, 67% among gMDSCs and 70% among mMDSCs.

To verify whether any specific pattern of [Ca^2+^]_i_ was correlated with any specific myeloid cell subset, PBMCs from 3 donors were incubated with BxPC3-*SMAD4*+ CM for 96 hours to enrich the immunosuppressive populations before performing FACS sorting. Monocytes, DCs, gMDSCs and mMDSCs were collected, seeded, and cultured in NC media for 48 hours before [Ca^2+^]_i_ analysis. Figure 4 shows [Ca^2+^]_i_ in the four myeloid cellular subsets. Monocytes had both peaks and irregular [Ca^2+^]_i_ whereas DCs did not pulse. FACS sorted gMDSCs hardly adhered to coverslips glass slides and only two records were available, but both cells had a stable profile. Conversely mMDSCs were characterized by the presence of well defined regular peaks and by the absence of irregular [Ca^2+^]_i_. FACS sorted gMDSCs and mMDSCs were stained with May-Grünwald-Giemsa (Figure 4, bottom panels).

### The *SMAD4*-dependent Exo miRNAs 494-3p and 1260a induce intracellular calcium fluxes in PBMCs

**Figure 5:**
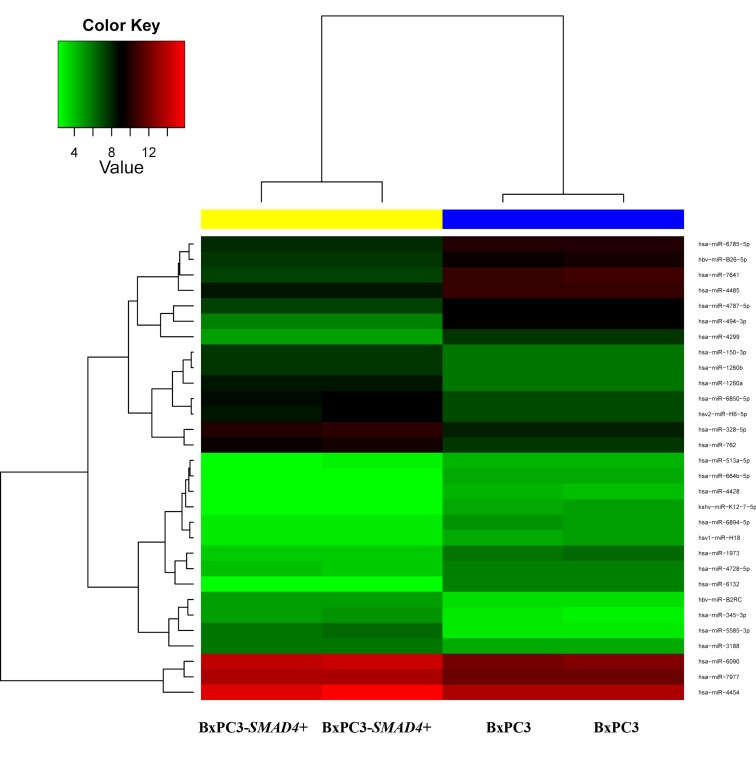
Heatmap for comparison between BxPC3-*SMAD4*+ and BxPC3 Exo miRNAs The color of the horizontal side bar at the top of the plot indicates the group membership of a given sample (yellow: BxPC3-*SMAD4*+, two replicates; blue: BxPC3, two replicates).

**Figure 6:**
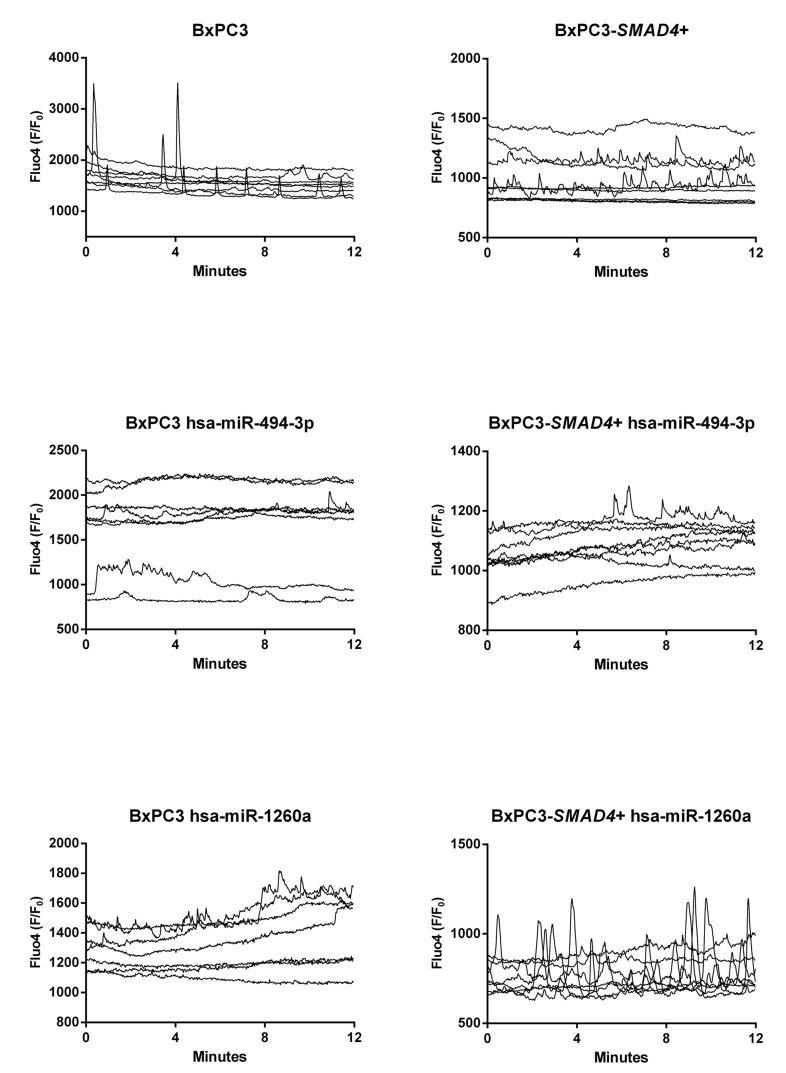
hsa-miR-494-3p and miR-1260a transfection alters intracellular calcium fluxes ([Ca^2+^]_i_ ) of PBMCs The variations in [Ca^2+^]_i_ of PBMCs after incubation for 72 hours in complete not transfected or hsa-miR-494-3p and hsa-miR-1260a transfected BxPC3 and BxPC3-*SMAD4*+ media are shown as Fluo4 F/F_0_ ratio. Each graph represents one experiment and each line [Ca^2+^]_i_ of one cell.

According to the microarray analysis, the heat map of the differentially expressed miRNAs was generated by unsupervised hierarchical clustering analysis (Figure 5). The most deregulated 30 miRNAs, detected by highest absolute value of logarithmized estimated fold change, are reported in Supplementary Table 4. Microarray data were validated using real time PCR, analyzing two of the most de-regulated miRNAs, hsa-miR-494-3p and hsa-miR-1260a. The Ct of three experimental replicates, each run in duplicate, obtained in BxPC3 derived Exo ranged from 30.93 to 31.91 for hsa-miR-494-3p and from 27.81 to 27.85 for hsa-miR-1260a. After normalization to U6 small RNA Exo content, the relative expression of any miRNA in BxPC3-*SMAD4*+ derived Exo was referred to that of BxPC3. It was confirmed that hsa-miR-494-3p was under expressed (relative quantification = 0.8±0.0, mean±SD), while hsa-miR-1260a was over expressed (1.2±0.0) in BxPC3-*SMAD4*+ with respect to BxPC3 derived Exo. To ascertain whether the differential expression of these two miRNAs was correlated with variations in calcium fluxes of myeloid cells, transfection induced expression and inhibition of hsa-miR-494-3p and of hsa-miR-1260a in both BxPC3 and BxPC3-*SMAD4*+ cells were performed. PBMCs were maintained in complete CM obtained from non transfected and transfected or inhibited pancreatic cancer cells. Figure 6 shows [Ca^2+^]_i_ of representative hsa-miR-494-3p and of hsa-miR-1260a transfection experiments, while Supplementary Figure 3 shows the corresponding findings with inhibition experiments. The induced expression of hsa-miR-494-3p in both pancreatic cancer cell lines and of hsa-miR-1260a in BxPC3-*SMAD4*+ cells determined in PBMCs a similar and progressive increase of [Ca^2+^]_i_. The magnitude of 10 minutes increment was calculated for any individual cell in all experimental conditions, being mean values confirmed to be significantly enhanced by hsa-miR-494-3p (Supplementary Figure 4, upper panels). hsa-miR-1260a caused a similar increment only when expressed by BxPC3. The expression or inhibition of these two miRNAs by BxPC3 did not affect the number of PBMCs with a peak behavior of [Ca^2+^]_i_ (Supplementary Figure 4, lower left panel). By contrast hsa-miR-1260a expression in BxPC3-*SMAD4*+ induced a significant increase in the percentage of PBMCs with regular calcium peaks (Supplementary Figure 4, lower right panel), and favored PBMCs to form clusters (Supplementary Figure 5).

### *SMAD4* loss determines enrichment in Exo proteins of the glycolysis biological process

**Figure 7:**
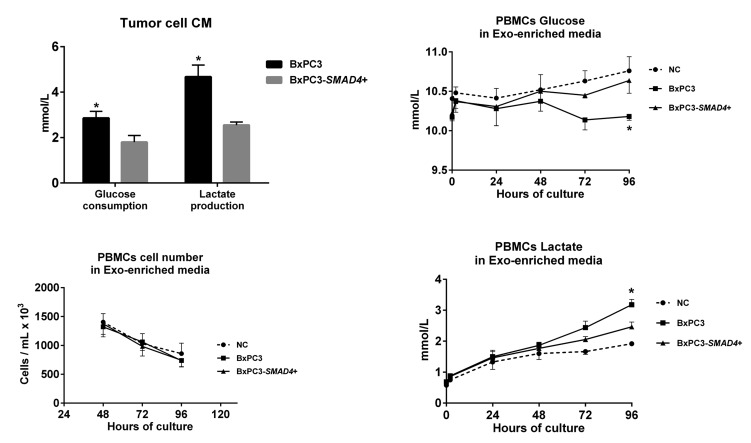
PDAC-derived Exo induce aerobic glycolysis in PBMCs Upper left panel: Glucose consumption and lactate production of BxPC3 and BxPC3-*SMAD4*+ cells (mean with standard deviation). The cells were cultured for 4 days in complete media 1% FCS to obtain conditioned media (CM). Glucose and lactate were measured in non conditioned and in CM from three separate experiments. For any experiment glucose consumption was calculated as the difference between glucose measured in non conditioned (11.5 mmol/L, mean of three replicates) and in CM. In non conditioned media lactate levels were always below the detection limit (<0.03 mmol/L), this supporting lactate levels measured in CM as lactate production. *Student’s t test for paired data: *t* = 12.95, *p*<0.01 for glucose consumption; *t* = 9.54, *p* = 0.01 for lactate production. Lower left panel: number of PBMCs cultured in Exo-enriched non conditioned (NC) medium and in Exo-enriched BxPC3 and BxPC3-*SMAD4*+ CM obtained as described in the upper left panel. Mean values (points) with standard deviation (bars) from three independent experiments, each in duplicate, are shown. Two-way Repeated measures analysis of variance: F = 14.16, *p* = 0.0012 for time effect; F = 0.72, p = 0.5101 for treatment effect; F = 0.2287, *p* = 0.9190 for time-treatment interaction. Upper and lower right panels: glucose and lactate measured in the supernatants of PBMCs cultured for 2, 24, 48, 72 and 96 hours in Exo-enriched NC and in Exo-enriched BxPC3 and BxPC3-*SMAD4*+ CM obtained as described in the upper left panel. Mean values (points) with standard deviation (bars) from three independent experiments, each in duplicate, are shown. Two-way Repeated measures analysis of variance: Time effect: F = 0.9175, *p* = 0.5077 for glucose and F = 49.68, *p*<0.0001 for lactate; Treatment effect: F = 9.943, *p* = 0.0280 for glucose and F = 10.11, *p* = 0.0273 for lactate; Time-treatment interaction: F = 2.420, *p* = 0.0444 for glucose and F = 8.997, *p*<0.0001 for lactate. *Tukey’s multiple comparison test: *p* < 0.05 with respect to NC PBMCs.

**Figure 8:**
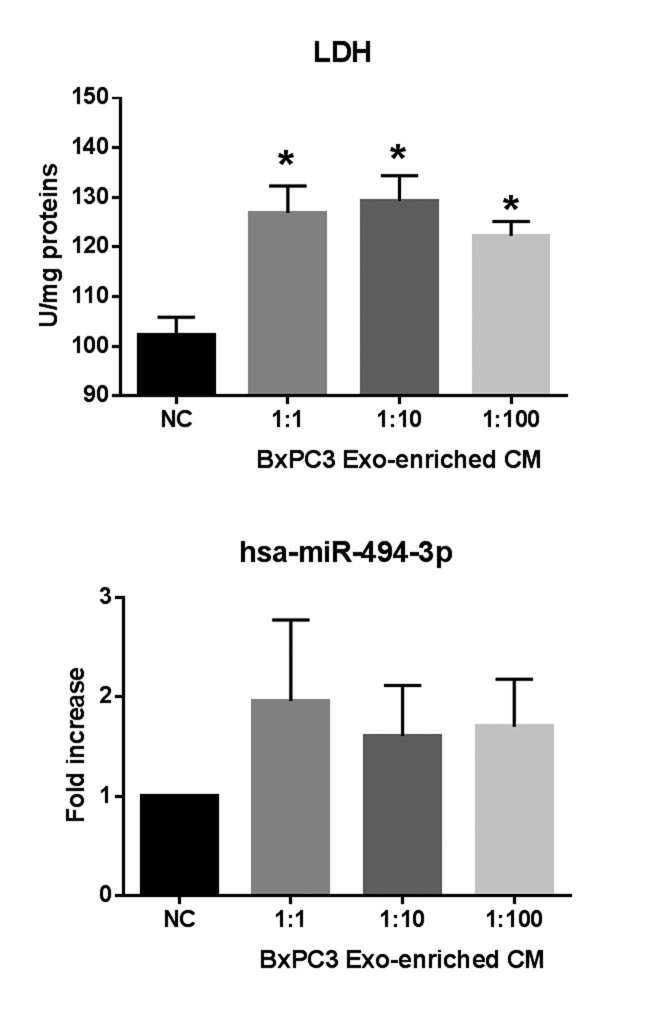
PDAC-derived Exo transfer LDH enzyme activity and hsa-miR-494-3p into PBMCs Upper panel: LDH activity in PBMCs after they have been cultured in the absence (NC) or in the presence of serially diluted BxPC3 derived Exo for 24 hours. Mean values (columns) with standard deviation (bars) from four independent experiments are shown. *Bonferroni’s test for pairwise comparison: *p* < 0.05 with respect to NC. Lower panel: hsa-miR-494-3p relative expression in PBMCs after they have been cultured in the absence (NC) or in the presence of serially diluted BxPC3 derived Exo for 24 hours. Mean values (columns) with standard deviation (bars) from four independent experiments are shown.

The differentially expressed proteins between BxPC3-*SMAD4*+ and BxPC3 derived Exo, evaluated by SILAC experiments, were defined as proteins with a ratio between heavy (BxPC3) and light (BxPC3-*SMAD4*+) isoforms below 0.667 and above 1.5, respectively. The identified proteins with a ratio below 0.667 were those under-expressed, while those with a ratio above 1.5 were over-expressed in BxPC3 with respect to BxPC3-*SMAD4*+ Exo. Supplementary Table 5 reports the Uniprot accession number, protein description, and heavy/light ratio of the *SMAD4*-related differentially expressed proteins in tumor-derived Exo. Using David software, a comprehensive analysis was made of differentially expressed proteins between BxPC3 and BxPC3-*SMAD4*+ derived Exo. The GO-terms for biological processes found to be significant after Benjamini and Hochberg adjustment for multiple comparisons were selected (Supplementary Figure 6). Eleven biological processes were identified, including translation, cell adhesion, cell signaling and glycolysis. Focusing on glycolysis, we found that BxPC3 cells had a higher degree of glucose consumption and of lactate production than BxPC3-*SMAD4*+ cells (Figure 7, upper left panel). To verify whether the different expression of glycolytic enzymes between BxPC3 and BxPC3-*SMAD4*+ derived Exo had an impact on glucose metabolism of myeloid cells, glucose and lactate were assayed in the supernatants of PBMCs cultured for 2, 24, 48, 72 and 96 hours in Exo-enriched CM and NC media, the results from three independent experiments each made in duplicate being shown in the right panels of Figure 7. BxPC3, not BxPC3-*SMAD4*+, Exo enriched media induced significantly greater glucose consumption and lactate production with respect to Exo enriched NC media. To verify whether PDAC-derived Exo transfer the glycolytic enzymes into PBMCs, the activity of LDH, one of the most differentially expressed proteins between BxPC3 and BxPC3-*SMAD4+* derived Exo, was assayed in PBMCs after they have been cultured for 24 hours in NC and in serially diluted BxPC3 Exo-enriched CM. LDH activity in PBMCs increased in the presence of Exo with respect to NC (Figure 8, upper panel: F = 7.777, *p* = 0.0093 from three independent experiments). Similarly, the expression levels of hsa-miR-494-3p were increased in PBMCs after they have been treated with BxPC3 Exo enriched CM, although differences were not statistically significant (Figure 8, lower panel: F = 0.568, *p* = 0.665). Transfection induced expression of hsa-miR-1260a, not of hsa-miR-494-3p, reduced glucose consumption and lactate production in BxPC3-*SMAD4*+ cells (F = 4.253, *P* = 0.009 and F = 4.751, *p* = 0.006).

## DISCUSSION

Alterations in genetic and epigenetic, transcription and signaling programs converge and concur in determining PDAC, a tumor expected to become the second most common cause of cancer-related mortality in 2030 in the absence of advances in therapy [36]. A relevant co-determinant in PDAC progression is the immunosuppressive ground associated with this tumor type [3]. In order to gain a deeper understanding of the complexity of immunoregulation in PDAC, in this study we provide evidence that *SMAD4* loss imbalances immature myeloid cellular subsets in favor of MDSCs by altering intracellular calcium doing this through miRNAs and proteins Exo transfer from cancer cells to myeloid cells.

We first observed that, in the presence of PDAC CM, T cells subsets were unbalanced towards immunosuppression: CD8^+^ T immunoeffector cells were reduced while CD4^+^CD25^+^ cells underwent expansion. These “in vitro” results are is in agreement with the decreased CD8^+^ and increased CD4^+^CD25^+^Foxp3^+^ T cell stromal infiltration found in human tumors, and described to be associated with shorter survival [18, 37]. Interestingly, these PDAC-driven effects on T cells were *SMAD*4-dependent, being observed when BxPC3 cells were forced to express *SMAD*4. The immunosuppressive T cell phenotype induced by PDAC CM might be mediated by soluble factors but also by factors carried in Exo, currently considered an important route for cell to cell communication [32, 38-40]. Both the soluble fraction and the Exo enriched fractions of PDAC CM failed to reproduce the effects on T cells evoked by complete media. This might alternatively or additionally depend on: 1. co-operation between soluble factors and Exo being required to alter T cells phenotypes; 2. magnitude of observed variations in T cells, subsets being too small to be reproduced by any single fraction. Moreover, PDAC-derived factors (e.g. GM-CSF) may directly trigger the variations observed in T cells phenotypes [20], but these variations are more likely to be secondarily induced by myeloid cells, a potential main target of PDAC-derived immunomodulatory factors [23, 41]. In line with this hypothesis, PDAC CM significantly altered myeloid cell subsets in a *SMAD*4 independent manner, causing the reduction of DCs and of their main cytokine, TNF, in favor of the induction of mMDSCs. gMDSCs, unlike mMDSCs, were inhibited by PDAC CM and this fits well with the hypothesized equilibrium between these two immunosuppressive myeloid cell populations, i.e. the expansion of one set being correlated with the inhibition of the other set [20]. Differently form complete media, Exo were able to induce the expansion of both mMDSC and gMDSC subsets, being these effects *SMAD*4 - related.

Since myeloid cells with an immunosuppressive phenotype prevailed in PDAC CM, we verified whether immunosuppressive cytokines increased in this setting. IL-1β, IL-4, IL-6 and IL10 were below the respective limits of detection, while TGF-β1 increased in PBMCs culture media on the fourth day of culture (2 fold) [20, 41]. However, no significant differences were found between NC and PDAC conditioned PBMCs. This finding might not support the hypothesis that TGF-β1 is associated with PDAC induced immunosuppression, but it might also be consequent to 1) the autocrine/paracrine action of this cytokine, the amount produced being rapidly taken up by producing and/or adjacent cells thus limiting the cytokine quota released in culture media and 2) our results come from one particular cell line which definitively does not represent the whole complexity of PDAC immunosuppressive networtk. In this network macrophages should also be considered, because they might enhance tumor-initiating capacity and suppress CD8^+^ T cells when educated by tumor cells [19], although in our experimental model macrophages appeared unaffected. This finding *per se* does not contradict the established concept that in PDAC tissue TAM increase and concur in enhancing tumor progression and chemoresistance. Our experimental model allows to investigate the effects of tumor derived soluble products and Exo on peripheral blood mononuclear cells, the precursors of TAM, which complete differentiation requires a more intimate and complex contact of monocytes with tumor cells and other stromal cells [22].

PDAC CM and Exo in particular might induce the expansion of immunosuppressive myeloid cells by activating critical intracellular signaling pathways, mainly JAK/STAT kinase, TGF-β and RAGE [25]. Calcium calmodulin is a critical pathway involved in many cellular functions, including myeloid cell differentiation [35, 42]. Regular [Ca^2+^]_i_ pulses are required to maintain normal myeloid cellular function, and any disruption of this mechanism might underlie alterations in phenotype and function such as those observed in the present study [43]. This was the premise of our subsequent experiments conducted to evaluate [Ca^2+^]_i_ fluxes in the above described experimental settings. PBMCs cultured in NC medium showed irregular variations in [Ca^2+^]_i_ resembling background noise. A higher frequency of [Ca^2+^]_i_ spikes were found in PBMCs cultured in BxPC3-*SMAD*4+, but mainly in BxPC3 complete CM, and these variation were reproduced by Exo enriched media, suggesting that in case of *SMAD4* loss cancer derived Exo magnify their ability to induce an increased calcium trafficking in PBMCs. Ca^2+^ responsible for this increased cellular activity might derive from the extra-cellular space and the intracellular stores [42]. We believe that both are involved, since after blocking membranal Ca^2+^ channels with verapamil, an overall reduction in [Ca^2+^]_i_ fluxes was observed but the difference between control and PDAC treated cells was maintained (data not shown). Since the variations in [Ca^2+^]_i_ pattern mirrored those in myeloid cell phenotypes, we analyzed the [Ca^2+^]_i_ pattern of single cells populations after they have been FACS sorted, and demonstrated that DCs are static while mMDSCs present regular and well defined [Ca^2+^]_i_ peaks. Only few FACS sorted gMDSCs could be analyzed, since they did not adhere to the glass supports and this might be consequent to increased apoptosis of sorted cells in standard media for 48 hours, in agreement with Stromnes et al. [20]. These data support the notion that PDAC cells and, in particular, PDAC derived Exo reduce DCs and expand mMDSCs through [Ca^2+^]_i_ changes, this phenomenon being *SMAD*4-dependent. This is the first time that such a mechanism has been described in literature.

Among the potential causative agents responsible for this pathogenic mechanism, *SMAD4*-related differences in the amount of Exo release might be hypothesized, also considering that a dose-related effect of BxPC3 derived Exo in inducing gMDSCs was found (data not shown). However, the role of *SMAD*4-differentially expressed miRNAs and/or proteins carried in PDAC-derived Exo, which transfer their cargo from cancer cells to immune cells thus altering their behavior should also be taken into account. Therefore we screened the differentially expressed miRNAs and proteins in BxPC3 and BxPC3-*SMAD4*+ derived Exo by microarray analysis and SILAC respectively.

A number of miRNAs were found to be differentially expressed between Exo from the two cell lines studied. We selected two of the most *SMAD4*-associated de-regulated miRNAs, the hsa-miR-494-3p, which was previously demonstrated by Li et al. to be closely associated with *SMAD4* expression [44], and hsa-miR-1260a, which association with *SMAD4* is reported here for the first time. To ascertain whether these two miRNAs have any role in the calcium balance of myeloid cells, transfection and inhibition experiments were performed. In the absence of *SMAD4* (BxPC3) the expression of both hsa-miR-494-3p and hsa-miR-1260a caused a progressive accumulation in intracellular calcium in PBMCs. The expression of hsa-miR-1260a augmented the percentage of PBMCs with regular calcium peaks and induced cells to form large clusters only when *SMAD4* was also expressed (BxPC3-*SMAD4*+), suggesting that these hsa-miR-1260a-related effects require other cancer derived *SMAD4*-related co-factors, being *SMAD4*-related proteins potential candidates. The most de-regulated proteins between *SMAD4* expressing and non expressing cellular derived Exo were mainly involved in translation, cell and matrix adhesion, integrin and Ras signaling, and this is in agreement with a more aggressive *SMAD4*-associated phenotype. Three proteins overexpressed in BxPC3 with respect to BxPC3-*SMAD4*+ derived Exo, appear of potential interest for further studies because of their known effects on immune cells and/or trans membrane ion fluxes: VDAC1, Versican and Spectrin beta [45, 46]. A number of glycolytic enzymes were overexpressed in BxPC3 than in BxPC3-*SMAD4*+ Exo, and glycolysis was one of the core biological processes significantly altered in an *SMAD4* associated manner and this allows us to add *SMAD4* to the repertoire of cancer-associated genetic alterations, which include *KRAS* activating mutations, *TP53* loss of function and *MYC* overexpression, which regulate the metabolic reprogramming of cancer cells, also known as the Warburg effect [47]. The *SMAD4* related enrichment of glycolytic enzymes in cancer derived Exo might underlie the reverse Warburg effect, the metabolic reprogramming involving stromal cells, such as cancer associated fibroblasts, cancer stem cells, and immune cells. We demonstrated that PDAC derived Exo transfer to myeloid cells the glycolytic enzyme activity of LDH, this finding being correlated with a direct and *SMAD4*-associated impact of Exo on myeloid cell glycolysis, since BxPC3 derived Exo caused a greater glucose consumption and lactate production than BxPC3-*SMAD4*+ Exo. However Exo might impact on cellular glucose metabolism not only through the transfer of glycolytic enzymes, but also because they might interfere with gene transcription and translation. miRNAs are candidate molecules in this context, and based on this we verified whether one of the most highly expressed miRNA in BxPC3-*SMAD4+* derived Exo, the hsa-miR-1260a, could be implicated in the regulation of glycolysis by transfection/inhibition experiments. The forced expression of this miRNA caused a reduced glucose consumption and lactate production in *SMAD4*-expressing cells suggesting that hsa-miR-1260a is an antagonist of the Warburg effect. The present study therefore demonstrates that metabolic reprogramming with increased glycolysis and conversion of glucose into lactate is *SMAD4* and hsa-miR-1260a dependent and that tumor cells can transfer the propensity to elevated glycolysis to stromal cells via Exo. This finding opens new ways for understanding the relationship between cancer metabolism and immune suppression recently reported to be dependent on the metabolic competition between tumor and immune cells [48].

In conclusion, *SMAD4* deletion might underlie a more aggressive PDAC phenotype through Exo transfer to immune cells of proteins and miRNAs that, by altering intracellular calcium and glycolysis, may cause the expansion of immunosuppressive myeloid cells.

## MATERIALS AND METHODS

### Cell lines

The pancreatic cancer cell lines BxPC3, known to carry a homozygous deletion of the *SMAD*4/DPC4 gene, and BxPC3-*SMAD4*+, obtained from the BxPC3 cells stably transfected with the pBK-cytomegalovirus (CMV)-*SMAD*4/DPC4 expression vector, were used. The characterization of the cellular model including the validation of the transfection efficacy has been described by us elsewhere [49]. Culture details are reported in Supplementary Methods.

### Exo enrichment for cellular experiments

Non conditioned media, BxPC3 and BxPC3-*SMAD4*+ conditioned media (CM), all containing 1% FCS, were enriched in Exo following the differential ultracentrifugation procedure detailed in Supplementary Methods.

### Isolation of human peripheral blood mononuclear cells (PBMCs)

Human PBMCs were isolated from blood donors’ buffy coats by differential density gradient centrifugation (Histopaque^®^-1077, Sigma-Aldrich, Milano, Italy, F/H). After being washed twice with saline solution to remove contaminating platelets and centrifuged at 1,200 rpm for 10 minutes, PBMCs were treated with a hemolysis solution (NH_4_Cl, KHCO_3_, EDTA Na_4_) for 10 minutes, centrifuged at 1, 200 rpm for 10 minutes and finally used for the experiments.

### Experimental design

PBMCs were cultured in complete, Exo- free and Exo-enriched CM and non conditioned media. After 4 culture days, PBMCs were analyzed by flow cytometry and cytokines were measured in the supernatants. Intracellular calcium fluxes were analyzed in the same experimental conditions after 3 culture days by epifluorescence (Fluo4).

### Flow cytometry analysis

Human PBMCs from 8 blood donor’s buffy coats were seeded in a six well culture plate (6x10^6^ in each well) and kept in culture for 96 hours with 3 mL of complete, Exo-free and Exo-enriched non conditioned, BXPC3 CM and BxPC3-*SMAD*4+ CM reconstituted with 10% FCS. After 96 hours, PBMCs were collected by scraping, and analyzed by flow cytometry as detailed in Supplementary Methods.

### Cell sorting

20x10^6^ PBMCs were cultured for 48h in 100 mm culture dishes in 10 ml of BxPC3-*SMAD4*+ CM. At least 20 culture dishes were prepared. After collection by scraping, 200x10^6^ PBMCs were incubated in the dark for 30 minutes with the following monoclonal antibodies (Beckman Coulter, Miami, FL, USA): 110 µL HLA-DR-PC5, 80 µL CD14-PC7, 80 µL CD45-ECD and 80 µL CD11b PE. Afterwards, PBMCs were washed with PBS containing 2% FCS, centrifuged at 1200 rpm for 10 minutes and re-suspended with PBS containing 2% FCS to obtain 15x10^6^ PBMCs per mL. The above-described four mononuclear cell subsets were FACS-sorted (BD FACSAria III, BD Biosciences, San Jose, CA, USA). Sorted cells were seeded on coverslips that had been inserted within each well of six well culture plates and cultured for 48 hours in 2 ml of complete control medium before [Ca^2+^]_i_ fluxes study. After [Ca^2+^]_i_ fluxes study, each coverslip was also stained in May-Grünwald-Giemsa. This experiment was repeated three times with buffy coats from three blood donors.

### Intracellular calcium fluxes analysis

2x10^6^ PBMC were seeded on coverslips that had been inserted in each well of six well culture plates and cultured for 72 hours in 2 ml of complete or Exo-enriched control, BxPC3 CM, BxPC3-*SMAD*4+ CM reconstituted at 10% FCS. Coverslips were then processed for the [Ca^2+^]_i_ fluxes study as previously described [49], using the intracellular calcium tracer Fluo-4AM (Invitrogen S.R.L.) at 5 μM. Three independent experiments, each made in triplicate, were performed.

### Cytokines assay

After centrifugation at 1,200 rpm for 10 minutes of PBMCs supernatants after 96 hours culture in the above-described conditions IL-1β, IL-4, IL-6, IL-10, TGF-β1 and TNF were analyzed by chemiluminescent immunometric assays (Immulite, Siemens Healthcare Diagnostic, UK) according to the manufacturer’s specifications. For all the experimental conditions, at least three independent sets of experiments were performed.

### Glucose, lactate and lactate dehydrogenase (LDH) assays

Glucose and lactate in culture supernatants were measured on the automated ARCHITECT System following the manufacturer’s instructions (Abbott Laboratories, Abbott Park, IL, USA). LDH enzymatic activity was determined in PBMCs lysates by using the automated COBAS System according to the manufacturer’s instructions (Roche Molecular Diagnostics, Pleasanton, CA, USA).

### miRNAs microarray analysis

The miRNAs expression profiling of BxPC3 and BxPC3-*SMAD4*+ CM Exo was performed in duplicate samples using the Agilent SurePrintG3 Human miRNA (8x60K) microarrays (custom design by Comprehensive Biomarker Center, CBC, recently re-named to Hummingbird Diagnostics GmbH, Heidelberg, Germany) as detailed in Supplementary Methods.

### miRNA transfection and inhibition experiments

Human hsa-miR-494-3p and hsa-miR-1260a were transfected (miRIDIAN mimic) and silenced (miRIDIAN Hairpin inhibitor)(Dharmacon, GE Healthcare, UK) in BxPC3 and BxPC3-*SMAD4*+ cells following the manufacturer’s instruction.

### SILAC experiment

BxPC3 and BxPC3-*SMAD4*+ cell lines were cultured in RPMI 1640 MEDIA FOR SILAC with 10% dialyzed fetal bovine serum (FBS), with the addition of either with the non-labelled aminoacids Lysin and Arginine (light medium) or with the labelled ^13^C_6_-Lysine and ^13^C_6_^15^N_4_-arginine (heavy medium) (Chemical Research 2000 srl, Rome, Italy). In a second experiment, the same cell lines were cultured by swapping media, BxPC3 being maintained in heavy medium and BxPC3-*SMAD4*+ maintained in light medium, thus creating two biological replicates of the same experiment. After eight days, cell media were changed with fresh media prepared as specified above but without the serum addition, in order to reduce any contaminant in the proteomic analyses, cells being cultured in this condition for 16 hours before media collection and exosomes enrichment made by ultracentrifugation (Beckman Coulter L-80XP Ultracentrifuge, Type 70 Ti rotor) as detailed in supplementary methods. The ratios for light/heavy and heavy/light were calculated for each identified protein of the two experiments. Proteins were considered significantly altered if the average value, calculated for the two ratios, was above 1.5 or below 0.667

### Statistical analysis

The statistical analysis of data was made by Repeated measures ANOVA, Tukey’s multiple comparisons test, Student’s t test for paired data and the chi square test using the GraphPad prism software (ver 6.04).

## SUPPLEMENTARY MATERIALS FIGURES AND TABLES















## Abbreviations

CM: conditioned media
DCs: dendritic cells
Exo: exosomes
FACS: Fluorescence-activated cell sorting
GM-CSF: granulocyte-macrophage colony-stimulating factor
IL: interleukin
LDH: lactate dehydrogenase
MDSCs: myeloid derived suppressor cells
PanINs: pancreatic intraepithelial neoplasms
PBMCs: peripheral blood mononuclear cells
PDAC: Pancreatic ductal adenocarcinoma
TAM: tumor associated macrophages
TGF: transforming growth factor
TNF: tumor necrosis factor
VDAC: voltage dependent anion selective channel protein
