# Supplementary material for: PDAC-derived exosomes enrich the microenvironment in MDSCs in a SMAD4-dependent manner through a new calcium related axis
Source: Oncotarget. 2017 Sep 13;8(49):84928–44. doi: 10.18632/oncotarget.20863 (PMC5689584; doi:10.18632/oncotarget.20863)
Supplement: Supplementary file 1 [file oncotarget-08-84928-s001.pdf]

# PDAC-derived exosomes enrich the microenvironment in MDSCs in a *SMAD4*-dependent manner through a new calcium related axis

## SUPPLEMENTARY MATERIALS

### Cell lines

The pancreatic cancer cell line BxPC3, kindly donated by Dr Andrea Galli (University of Florence, Italy), was maintained in continuous culture in RPMI supplemented with 10% Fetal Calf Serum (FCS), 1% L-glutamine, 0.1% gentamycin and fungizone 0.25µg/mL (all reagents from Gibco/BRL, Gaithhersburg, MD, USA). This cell line is known to carry a homozygous deletion of the *SMAD*4/DPC4 gene. The BxPC3-*SMAD4*+ cell line was obtained from the BxPC3 cells stably transfected with the pBK-cytomegalovirus (CMV)-*SMAD*4/DPC4 expression vector as described by us elsewhere [36]. To obtain conditioned media (CM), $2\times10^5$ or $5x10^6$ BxPC3 and BxPC3-*SMAD4*+ were seeded in 75-$cm^2$ flasks with 15 or 10 mL RPMI with 1% FCS for FACS or calcium fluxes experiments, respectively, and kept in continuous culture at 37 °C in a humidified atmosphere (5% $CO_2$) for four days. CM were collected, centrifuged at 1,200 rpm for 10 minutes, adjusted to 10% FCS, to guarantee PBMCs survival, and used for the experiments with PBMC within two hours from collection.

### Exo enrichment for cellular experiments

Ten mL non conditionedmedia, BxPC3 and BxPC3-*SMAD4*+ CM, all containing 1% FCS, were enriched in Exo following the differential ultracentrifugation procedure originally described by Raposo et al. (Suppl. Ref. 1). Briefly, complete media were centrifuged at 2,000g at 4 °C for 10 minutes to remove dead cells and large cell debris. Supernatants were centrifuged at 10,000g at 4 °C for 30 minutes to remove cell debris. The resulting supernatants were ultracentrifuged at 100,000g at 4°C for 60 minutes to obtain Exo (Beckman Coulter Optima™ MAX-E). The supernatants (Exo free) were collected while Exo-containing pellets were re-suspended in 9 mL complete fresh media (Exo enriched). Complete media, Exo free and Exo-enriched fractions were kept at 4 °C for no more than 5 hours before the experiments.

### Western blot analysis

Exo enrichment was verified by western blot analysis of Alix, a cytoplasmic protein required for vesicle formation. Exo free and Exo enriched fractions from control and CM as well as BxPC3 and BxPC3-*SMAD4*+ cells were lysed in 100 µL cold lysis buffer (20 mMTris– HCl, pH 7.5, 150 mMNaCl, 1 mM EDTA, 1% Triton X- 100, 50 mMNaF, 10 mM Na4P2O7, 1 mM Na3VO4, and 10% protease inhibitor cocktail (Sigma-Aldrich, Milano, Italy)). Lysates were centrifuged for 10 minutes at 16,000 x g at 4 °C to remove insoluble materials, and supernatants were collected for western blot analysis. Total proteins (10 µg, Qubit Protein Assay Kit, Molecular Probes, Life Technologies, Monza, Italy) were separated by electrophoresis using precast NuPAGENovex 4-12% Bis-Tris gels (Invitrogen Life Technologies, Monza, Italy) in MES running buffer at constant 150V for 1h. After electrophoresis, proteins were electrotransferred onto nitrocellulose membranes (iBlot® Transfer Stacks, Invitrogen Life Technologies, Monza, Italy) using the iBlot® Dry Blotting System (Invitrogen Life Technologies, Monza, Italy) and membranes incubated with blocking buffer (5% low-fat milk powder in PBS-T (PBS with 0.1% Tween-20) for 1h at RT. Membranes were probed overnight at 4°C with the primary antibody mouse anti-Alix (Santa Cruz Biotechnology Dallas, Texas, USA, 1:1000 in blocking buffer). Blots were washed three times in PBS-T for 15 minutes and incubated for 1 hour at 4 °C with horse anti-mouse IgG HRP-linked secondary antibody (Cell Signaling Technology, Danvers, MA, USA, 1:5000 in blocking buffer). Western blots were washed three times in PBS-T for 15 minutes and signals were detected using the ECL Advance Western Blot Detection Kit (GE Healthcare Technologies, Milano, Italy). Supplementary Fig. 7 shows western blot of Alix in whole cell lysates (positive control), Exo enriched andExo free media of a representative experiment.

## Flow Cytometry Analysis

Human PBMCs from 8 blood donor's buffy coats were seeded in a six well culture plate (6x$10^6$ in each well) and kept in culture for 96 hours with 3 mL of complete, Exo-free and Exo-enriched non conditioned, BXPC3 CM and BxPC3-*SMAD*4+ CM reconstituted with 10% FCS. After 96 hours, PBMCs were collected by scraping, and analyzed by flow cytometry as previously described (Ref. N 16). Lymphocyte and immature myeloid cells subsets were studied using the antibodies described in Supplementary Table 6, with the following panels: CD4, CD8, CD3, CD45 for detecting of CD4$^+$ and CD8$^+$ T cells; CD4, CD3, CD25, CD45 for detection of CD4$^+$CD25$^+$ lymphocytes; CD11b, HLA-DR, CD45, CD14 for detecting of immature myeloid cells, which were classified as Monocytes (CD11b$^+$CD14$^+$HLA-DR$^+$), mMDSCs (CD11b$^+$CD14$^+$HLA-DR$^-$), gMDSCs (CD11b$^+$CD14$^-$HLA-DR$^-$), Dendritic Cells (CD11b$^+$CD14$^-$HLA-DR$^+$); CD11b, CD45, HLA-DR, CD14, CD16, CD80, CD86 for macrophages and dendritic cells. Immunophenotyping was performed by 10-colors flow cytometer Navios (Beckman Coulter, Miami, FL, USA). Acquisition was run until 100,000 events. Data analyses were made with Kaluza Software (Beckman Coulter, Miami, FL, USA). Apoptosis was evaluated by flow cytometry using the Annexin V Fluos Staining kit (Roche Diagnostics GmbH, Penzberg, Germany).

## miRNA microarray analysis

The CBC custom content Agilent miRNA slides contain all miRNAs from the Sanger miRBase release 19. Each glass slide is formatted with 8 high-definition 60K arrays (8x60k design / 8 arrays with 60 000 features each). Agilent human miRNA microarray labeling, washing and detection were performed following the manufacturer's standard protocols. Microarrays were analyzed using the Latest High-Resolution Microarray Scanner GS2505_C and array images to get raw data were analyzed by the Feature Extraction software (version 10.7.3.1, Agilent Technologies). Background correction, quantile normalization, and summarization of intensities were performed by the RMA (robust multiarray average) algorithm. The statistical analysis for identifying differentially expressed miRNA was performed by correlation, MA-plot, hierarchical clustering, heatmap, principal component analysis (PCA), t-test, fold change and empirical Bayes statistics. The threshold set for up- and down-regulated miRNA was a logarithmized fold change ≥1 and a ≤0.05 P value. Validation of over and under-expressed miRNA was performed by real time PCR (ABI Prism 7900HT, Applied Biosystems, Waltham, MA, USA) by the analysis of hsa-miR-494-3p and hsa-miR-1260a, performed in three replicated experiments. miRNA was extracted by miRCURY™ RNA Isolation Kit – Cell & Plant (EXIQON, Vedbaek, Denmark). RNA was quantified (Quant-It RiboGreen RNA Reagent and Kits, Life Technologies) and 12 ng were reverse transcribed by Taqman MicroRNA Reverse Transcription Kit (Applied Byosistems) and then PCR amplified (Taqman microRNA Assays, ID: 001973. for U6 (small nuclear RNA); ID: 002365 for hsa-miRNA 494-3p; ID:002896 for hsa-miR-1260a)(Applied Biosystems).

## SILAC experiment

To obtain exosomesenrichement, seventy-five mL each media were centrifuged for 30 minutes at 16,500 x g, then supernatants were collected and centrifuged 70 minutes at 150,000 x g. After discarding the resulting supernatants, the pellets were washed once with cold PBS, re-suspended in 6 mL PBS and centrifuged for 70 minutes at 150,000 x g. After discarding the supernatants, the pellets were re-suspended in 100 µL cold lysis buffer [20 mMTris–HCl, pH 7.5, 150 mMNaCl, 1 mM EDTA, 1% Triton-X 100, 50 mMNaF, 10 mM $Na_4P_2O_7$,1mM $Na_3VO_4$, and 10% protease inhibitor cocktail (Sigma Aldrich SRL, Milano, Italy)]. Total proteins in Exo enriched media were measured using the Bio-Rad protein assay (Bio-Rad Laboratories, Milano, Italy). Exo enriched proteins from heavy (15.1 µg) and light (15.1 µg) experiments were mixed, loaded onto a precast gel (NuPAGE, 4-12% Bis-Tris, Invitrogen) and electrophoresed. Each electrophoretic lane was then divided into four slices which then underwent reduction/alkylation and in-gel digestion with sequencing grade modified trypsin (Promega) as described elsewhere (Suppl. Ref. 2). Peptides were extracted from the gel by three changes of 50% acetonitrile/0.1% formic acid (FA). Samples were dried under vacuum, suspended in 3% acetonitrile/0.1% FA and loaded into a 10 cm pico-frit column (75 um I.D., 15 um Tip, New Objective) packed in-house with C18 material (Aeris Peptide 3.6 um XB-C18, Phenomenex). Peptides were separated with a HPLC Ultimate 3000 (Dionex – Thermo Fisher Scientific) using a linear gradient from 3 to 50% of acetonitrile/0.1 FA in 90 min at a flow rate of 250 nL/min. LC-MS/MS analysis was conducted with a LTQ-Orbitrap XL mass spectrometer (Thermo Fisher Scientific) as described (Suppl. Ref. 3). Data were analyzed with the Proteome Discoverer software (version 1.4, Thermo Fisher Scientific) interfaced to a Mascot server (version 2.2.4, Matrix Science) and searched against the human section of the Uniprot Database (www.uniprot.org, version 20150401, 90411 sequences) using carbamidomethyl cysteine for static modification and $^{13}C_6$-Lysine, $^{13}C_6{}^{15}N_4$-arginine, and methionine oxidation for variable modifications. Precursor and fragment tolerance were set at 10 ppm and 0.6 Da, respectively. Samples were searched using a

MudPIT protocol and the algorithm Percolator was used to calculate False Discovery Rate (FDR) based on the search against a randomized database. The results were filtered in order to consider only proteins identified with at least two unique peptides and high confidence ($q < 0.01$). Only unique peptides were considered for quantification.

### Exo transfer from PDAC to PBMCs

Exo enriched media were obtained, as described above, from a huge number of cancer cells ($50x10^6$ BxPC3) were seeded in 75-$cm^2$ flasks (n=10) with 10 mL RPMI with 1% FCS.

PBMCs from 2 blood donors buffy coat were cultured for 24 hours in non conditioned media (NC) and in whole (BxPC3 conditioned media 1:1) and serially diluted (1:10 and 1:100) Exo enriched media.

The expression levels of miR-494-3p were evaluated by real time PCR (as described above) in PBMCs lysates after 24 hours of culture.

## SUPPLEMENTARY REFERENCES


Suppl. Ref. 1. Raposo G, Nijman HW, Stoorvogel W, Liejendekker R, Harding CV, Melief CJ, Geuze HJ. B lymphocytes secrete antigen-presenting vesicles. J Exp Med 1996; 183:1161–72.

Suppl. Ref. 2. Arrigoni G, Pagano MA, Sarno S, Cesaro L, James P, Pinna LA. Mass spectrometry analysis of a protein kinase CK2beta subunit interactome isolated from mouse brain by affinity chromatography. J Proteome Res 2008; 7:990–1000.

Suppl. Ref. 3. Belluzzi E, Gonnelli A, Cirnaru M-D, Marte A, Plotegher N, Russo I, Civiero L, Cogo S, Carrion MP, Franchin C, et al. LRRK2 phosphorylates pre-synaptic N-ethylmaleimide sensitive fusion (NSF) protein enhancing its ATPase activity and SNARE complex disassembling rate. MolNeurodegener 2016; 11:1.


### For Supplementary Tables see in Supplementary Files

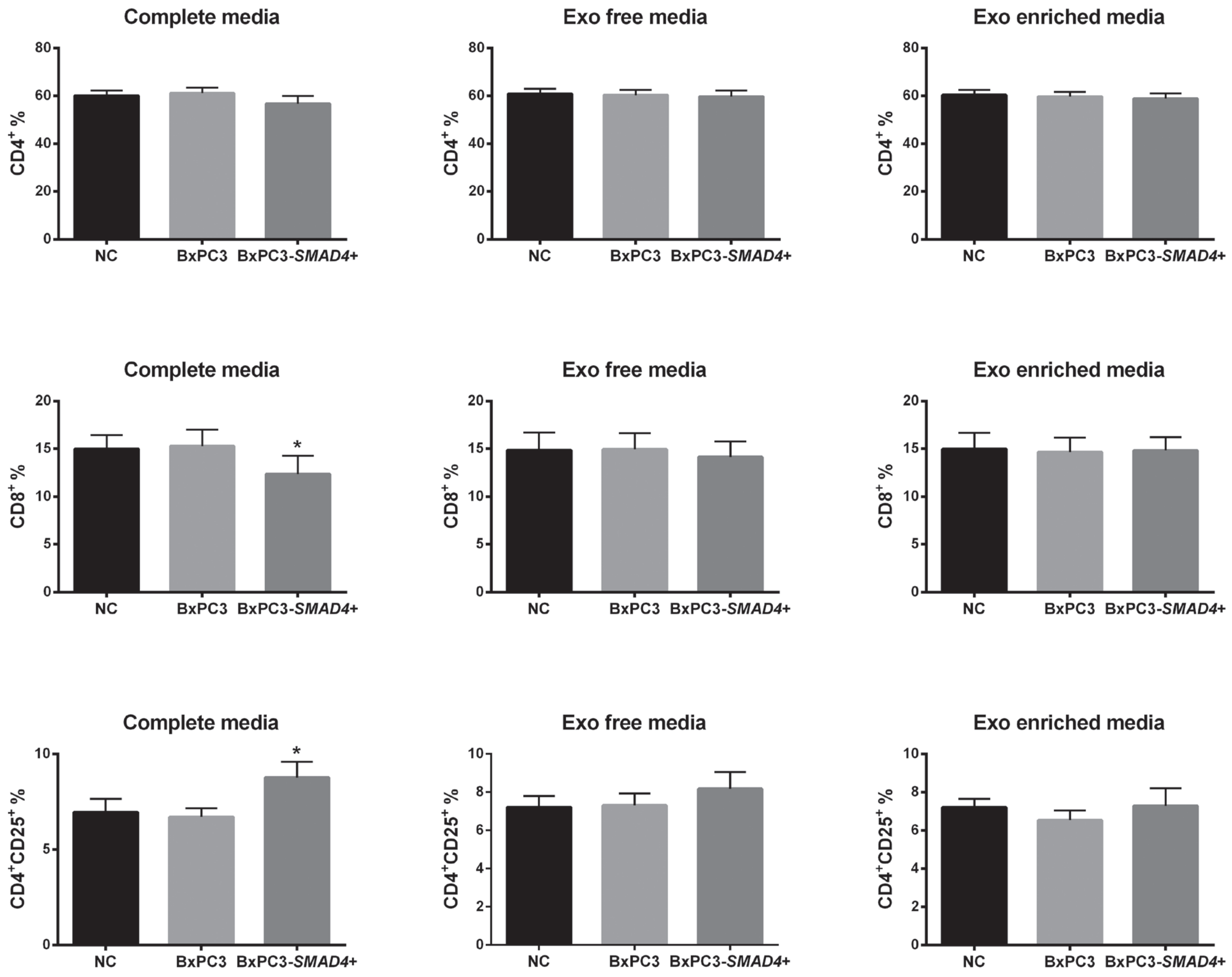


**Supplementary Figure 1: BxPC3-SMAD4+ CM expand $CD4^+CD25^+$ and reduces $CD8^+$ T cells.** $CD4^+$, $CD8^+$ and $CD4^+CD25^+$ T cells counted after PBMCs were incubated for four days with complete, Exo free and Exo enriched BxPC3 (dark grey columns) or BxPC3-*SMAD4+* (light grey columns) CM. PBMCsfrom each donor were run in parallel in complete, Exo free and Exo enriched non-conditioned culture medium (NC, black columns). All data are given as the average values ± SEM obtained from eight donors. *=p<0.05 with respect to NC and BxPC3 (Tukey's multiple comparisons test).

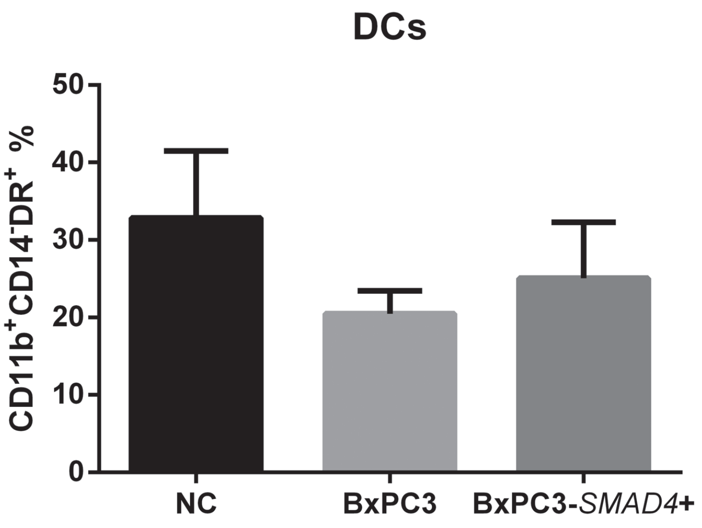


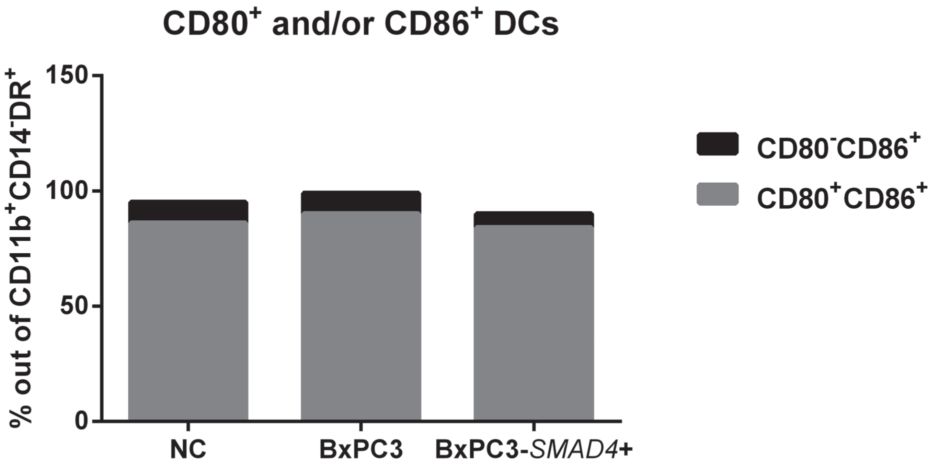


**Supplementary Figure 2: PDAC conditioned media reduce dendritic cells.** Upper panel: Mean values (columns) and standard deviations (bars) from two independent experiments showing Dendritic cells (DCs) identified as $CD11b^{+}CD14^{-}DR^{+}$ after PBMCs were incubated for four days with complete BxPC3 or BxPC3-*SMAD4+* conditioned media (CM). PBMCs were always run in parallel in complete non-conditioned culture medium (NC). One wayAnova: F=0.875, p=0.437. Lower panel: average of the percentage of $CD80^{-}CD86^{+}$ and of $CD80^{+}CD86^{+}$ cells among the dendritic cells shown in the upper panel.

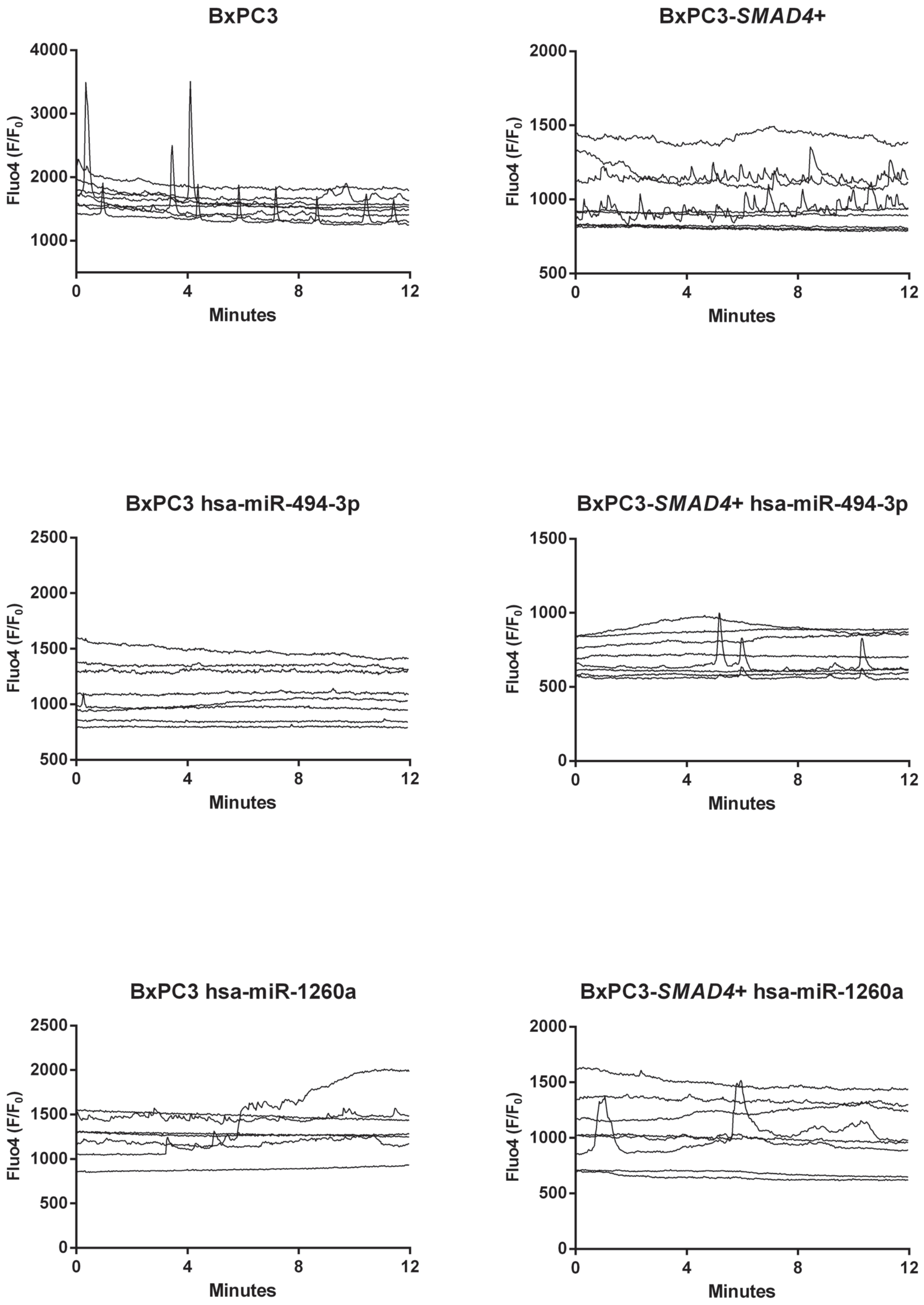


**Supplementary Figure 3: hsa-miR-494-3p and hsa-miR-1260a inhibition does not alter intracellular calcium fluxes ($[Ca^{2+}]_i$) of PBMCs.** The variations in $[Ca^{2+}]_i$ after PBMCs were incubated for 72 hours in complete conditioned media from BxPC3 and BxPC3-*SMAD4+*in which hsa-miR-494-3p and hsa-miR-1260a were silenced by miRIDIAN Hairpin inhibitor. The variations in $[Ca^{2+}]_i$ are shown as Fluo4 $F/F_0$ ratio. Each graph represents one experiment and each line $[Ca^{2+}]_i$ of one cell.

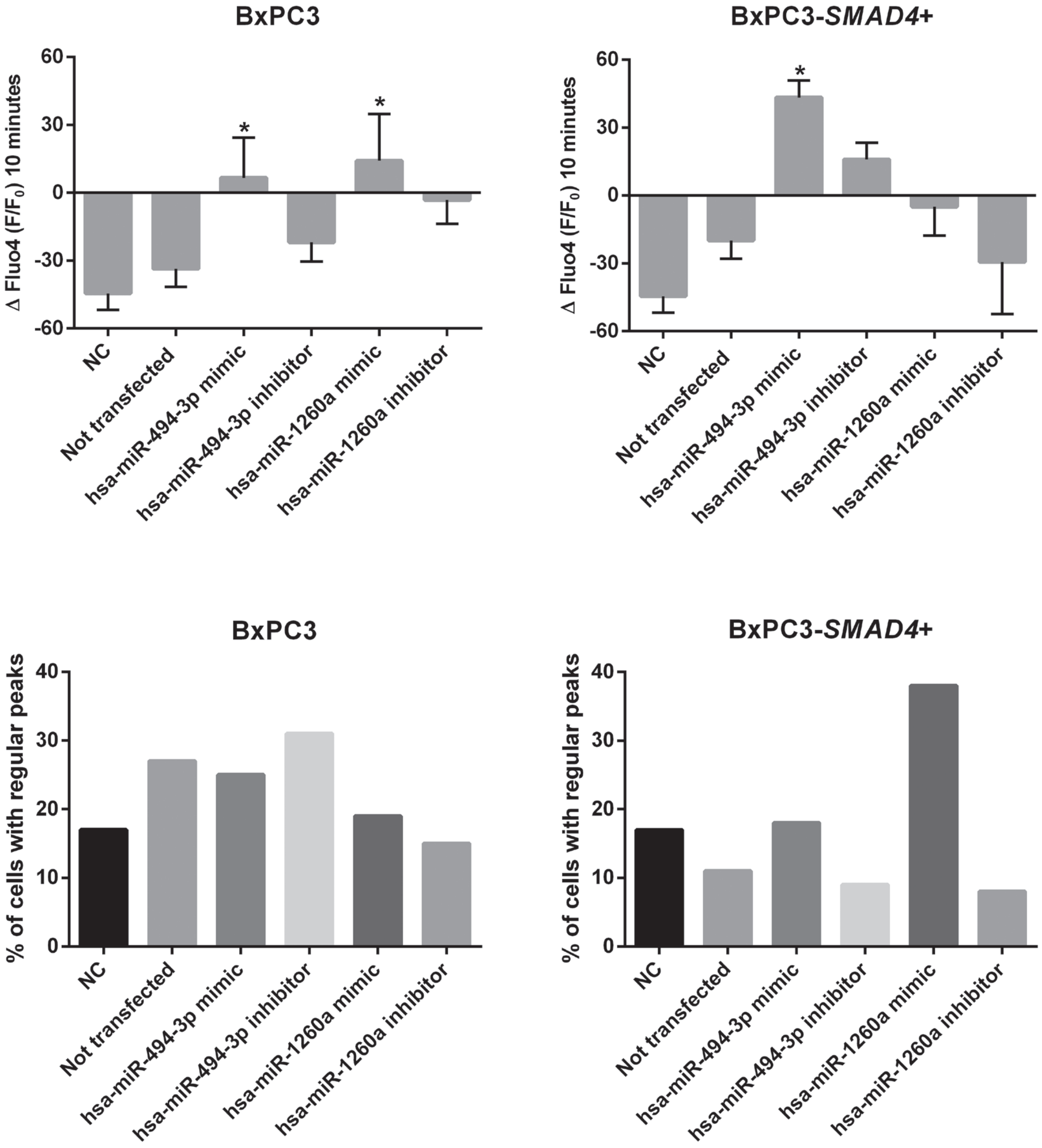


**Supplementary Figure 4: hsa-miR-494-3p and hsa-miR-1260a transfection induces intracellular calcium accumulation in PBMCs.** The upper graphs show the mean difference (columns) with standard errors (bars) in Fluo4 $F/F_0$ in a 10 minutes interval while the bottom graphs illustrate the percentage of cells with regular $[Ca^{2+}]_i$ peaks. Any individual cell was analyzed for the two considered parameters. A minimum of 22 cell data for any condition were obtained from 3independent experiments. One-way Anova for BxPC3 (upper left panel): F=3.456, p=0.0054; One-way Anova for BxPC3-*SMAD4*+ (upper right panel): F=11.65, p<0.0001. *=p<0.05 with respect to non conditioned PBMCs (NC); **=p<0.05 with respect to all the other conditions (Tukey's multiple comparison test). Chi-square test for BxPC3 (lower left panel): $X^2$=2.64, p=0.619; Chi-square test for BxPC3-*SMAD4*+ (lower right panel): $X^2$=10.07, p=0.039.

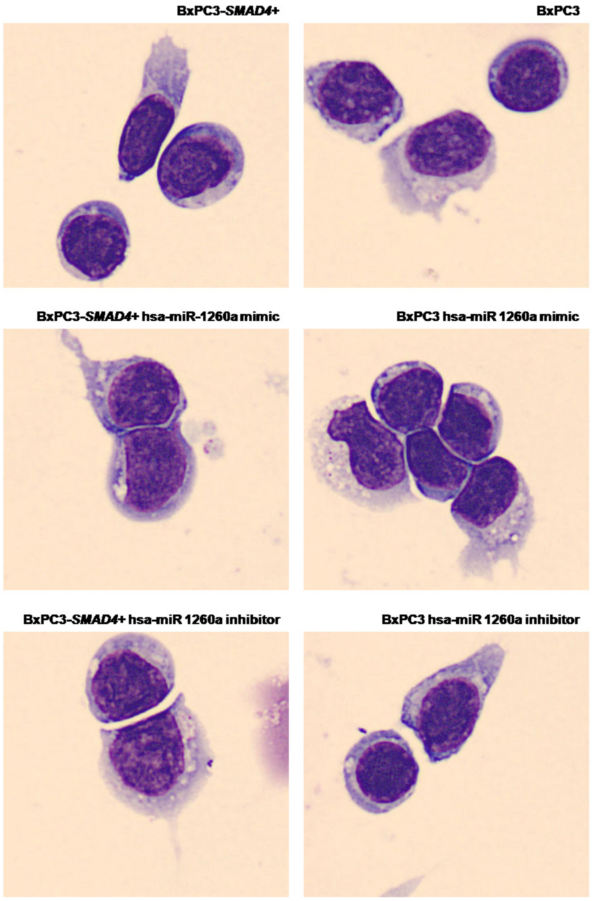


**Supplementary Figure 5: hsa-miR-1260a expression induce the formation of PBMCs clusters.** PBMCs were incubated for 72 hours in complete conditioned media from BxPC3 and BxPC3-*SMAD4*+in which hsa-miR-1260a was expressed or silenced by transfection (miRIDIAN mimic and miRIDIAN Hairpin inhibitor). Light microscopy (50×) after May-Grünwald-Giemsa staining.

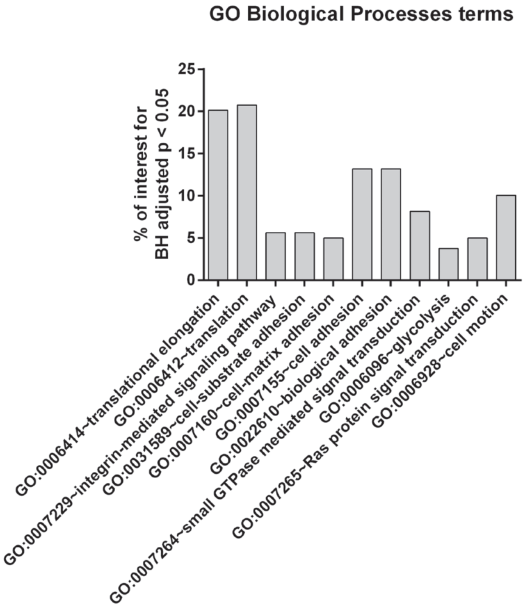


**Supplementary Figure 6: Pathways defined by *SMAD4* differentially expressed Exo proteins.** Biological pathways over-represented by *SMAD4*-related proteins differentially expressed in pancreatic cancer cells derived Exo. The y-axis is the percentage of interest of significant pathways (non-log p-value of 0.05 after Benjamini and Hochberg correction).

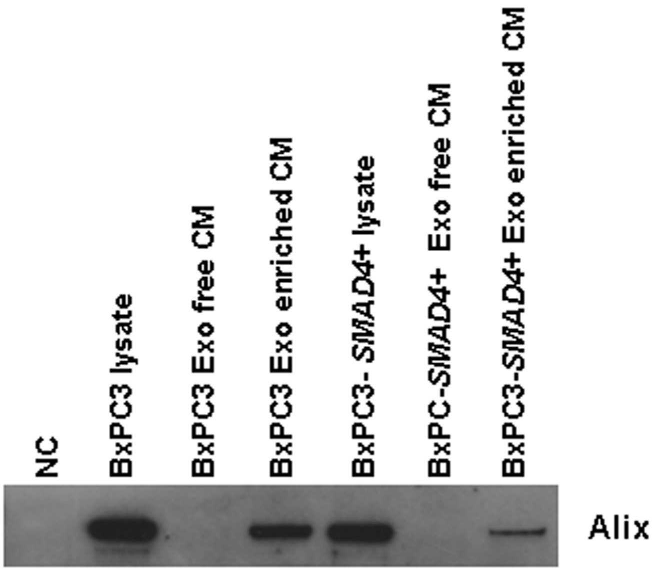


**Supplementary Figure 7: Western blot analysis of Alix.** Line 1 (NC): not conditioned complete medium (RPMI with 1% FCS). Lines 2 to 7: BxPC3 and BxPC3-*SMAD4*+ whole cell lysates, Exo enriched andExo free conditioned media (CM) obtained after four days of culture in RPMI 1% FCS.
