# Supplementary material for: PDAC-derived exosomes enrich the microenvironment in MDSCs in a SMAD4-dependent manner through a new calcium related axis
Source: Oncotarget. 2017 Sep 13;8(49):84928–44. doi: 10.18632/oncotarget.20863 (PMC5689584; doi:10.18632/oncotarget.20863)
Supplement: Supplementary file 2 [file oncotarget-08-84928-s002.docx]

**Supplementary Table 1.** Percentages of PBMCs positively stained for Annexin V and/or propidium iodine (PI) after four days in non conditioned, BxPC3 and BxPC3-*SMAD4*+ conditioned complete, Exo enriched and Exo free media. The percentages were calculated out of CD11b^+^ cells. Mean values and standard deviations (SD) obtained from two independent experiments were analyzed by the One-way Analysis of Variance (One way Anova).

|  |  | **Non conditioned media**  **Mean±SD (%)** | **BxPC3 CM**  **Mean±SD (%)** | **BxPC3-*SMAD4*+ CM**  **Mean±SD (%)** | **One way Anova** |
| --- | --- | --- | --- | --- | --- |
| **Annexin V-/PI-** | **Complete media** | 74.22**±**1.58 | 74.5**±**0.35 | 74.8**±**1.13 | F=0.131, p=0.882 |
|  | **Exo free media** | 74.5**±**2.03 | 74.5**±**0.23 | 76.0**±**0.35 | F=0.985, p=0.469 |
|  | **Exo enriched media** | 72.6**±**2.12 | 75.6**±**2.62 | 74.4**±**1.2 | F=1.03, p=0.456 |
| **Annexin V+/PI-** | **Complete media** | 16.9**±**2.22 | 16.4**±**1.20 | 15.8**±**1.56 | F=0.218, p=0.816 |
|  | **Exo free media** | 16.1**±**1.97 | 14.9**±**0.01 | 15.0**±**0.64 | F=0.615, p=0.597 |
|  | **Exo enriched media** | 17.3**±**2.34 | 15.0**±**2.84 | 16.2**±**1.98 | F=0.476, p=0.661 |
| **Annexin V+/PI+** | **Complete media** | 8.4**±**0.28 | 8.6**±**0.58 | 8.8**±**0.14 | F=0.551, p=0.626 |
|  | **Exo free media** | 8.8**±**0.33 | 9.5**±**0.26 | 8.4**±**0.21 | F=9.012, p=0.054 |
|  | **Exo enriched media** | 9.4**±**0.35 | 9.0**±**0.12 | 9.0**±**0.57 | F=0.557, p=0.623 |
| **Annexin V-/PI+** | **Complete media** | 0.50**±**0.28 | 0.58**±**0.25 | 0.66**±**0.51 | F=0.095, p=0.912 |
|  | **Exo free media** | 0.54**±**0.33 | 1.16**±**0.48 | 0.68**±**0.11 | F=1.829, p=0.303 |
|  | **Exo enriched media** | 0.41**±**0.29 | 0.55**±**0.06 | 0.45**±**0.21 | F=0.230, p=0.807 |
