# Supplementary material for: PDAC-derived exosomes enrich the microenvironment in MDSCs in a SMAD4-dependent manner through a new calcium related axis
Source: Oncotarget. 2017 Sep 13;8(49):84928–44. doi: 10.18632/oncotarget.20863 (PMC5689584; doi:10.18632/oncotarget.20863)
Supplement: Supplementary file 3 [file oncotarget-08-84928-s003.docx]

**Supplementary Table 2.** Percentages of monocytes derived macrophages (CD11b^+^CD16^+^) detected by flow cytometry in the monocytes and in the mMDSCs subsets after four days in non conditioned, BxPC3 and BxPC3-*SMAD4*+ conditioned complete, Exo enriched and Exo free media. The percentages of macrophages were calculated with respect to monocytes (HLA-DR^+^/CD14^+^) and mMDSCs (HLA-DR^-^/CD14^+^). Mean values and standard deviations (SD) obtained from two independent experiments were analyzed by the One-way Analysis of Variance (One way Anova).

| **Gating** |  | **CD11b^+^CD16^+^** | | | **One way Anova** |
| --- | --- | --- | --- | --- | --- |
|  |  | **Non conditioned media**  **Mean±SD (%)** | **BxPC3 CM**  **Mean±SD (%)** | **BxPC3-*SMAD4*+ CM**  **Mean±SD (%)** |  |
| **Monocytes** | **Complete media** | 83±9 | 89±7 | 91±4 | F=5.242, p=0.262 |
|  | **Exo free media** | 80±18 | 94±3 | 75±16 | F=0.673, p=0.563 |
|  | **Exo enriched media** | 80±18 | 89±6 | 88±5 | F=1.066, p=0.490 |
| **mMDSCs** | **Complete media** | 17±9 | 11±7 | 10±5 | F=6.379, p=0.240 |
|  | **Exo free media** | 19±18 | 5±2 | 16±5 | F=0.708, p=0.555 |
|  | **Exo enriched media** | 15±10 | 11±6 | 12±6 | F=1.525, p=0.433 |
