# Supplementary material for: PDAC-derived exosomes enrich the microenvironment in MDSCs in a SMAD4-dependent manner through a new calcium related axis
Source: Oncotarget. 2017 Sep 13;8(49):84928–44. doi: 10.18632/oncotarget.20863 (PMC5689584; doi:10.18632/oncotarget.20863)
Supplement: Supplementary file 4 [file oncotarget-08-84928-s004.docx]

**Supplementary Table 3. PDAC CM inhibit the TNF release from PBMCs.** The levels of TNF measured in PBMCs supernatants after four days in BxPC3 and BxPC3-*SMAD*4+ complete CM were expressed as percentage with respect to the corresponding levels of TNF of NC PBMCs, which were considered the reference 100% value for any donor (Ref., n=19). In a subset of 12/19 donors parallel experiments were performed using Exo enriched NC and CM, results being referred to the same reference. The statistical analysis was made by the One way Analysis of Variance (One way Anova).

|  | **Control**  **Mean±SEM (%)** | **BxPC3 CM**  **Mean±SEM (%)** | **BxPC3-*SMAD4*+ CM**  **Mean±SEM (%)** | **One-way Anova** |
| --- | --- | --- | --- | --- |
| **Complete media (n=19)** | 100 (Ref.) | 40.28±6.82# | 75.14±9.53* | F=19.68, p<0.0001 |
| **Exo enriched media (n=12)** | 227.60±51.80 | 242.5±65.84 | 186.00±51.09 | F=0.268, p=0.767 |

Tukey's multiple comparisons test: # = p<0.001 with respect to control and p<0.01 with respect to BxPC3-*SMAD4*+; * = p<0.05 with respect to control.
