# Supplementary material for: PDAC-derived exosomes enrich the microenvironment in MDSCs in a SMAD4-dependent manner through a new calcium related axis
Source: Oncotarget. 2017 Sep 13;8(49):84928–44. doi: 10.18632/oncotarget.20863 (PMC5689584; doi:10.18632/oncotarget.20863)
Supplement: Supplementary file 5 [file oncotarget-08-84928-s005.docx]

**Supplementary Table 4. *SMAD4* deregulated Exo miRNAs.** The most deregulated 30 probes (detected by highest absolute value of logarithmized estimated fold change) between BxPC3 and BxPC3-*SMAD4*+ derived Exo.

|  |  | Mean of normalized data (log intensities) | | Estimated fold change (FC) | Log_2_ FC | t-test | | AUC | Empirical Bayes limma | |
| --- | --- | --- | --- | --- | --- | --- | --- | --- | --- | --- |
|  |  | BxPC3-*SMAD4*+ | BxPC3 |  |  | Raw p | Adjusted p |  | Raw p | Adjusted p |
| *SMAD4*-associated down-regulated miRNAs | hsa-miR-494-3p | 5.69 | 9.22 | 0.09 | -3.53 | 2.82E-04 | 3.55E-02 | 1.00 | 4.06E-12 | 2.27E-09 |
|  | hsa-miR-7641 | 7.31 | 10.67 | 0.10 | -3.37 | 1.04E-03 | 5.35E-02 | 1.00 | 3.14E-11 | 5.91E-09 |
|  | hsa-miR-6132 | 2.44 | 5.70 | 0.10 | -3.26 | 3.14E-05 | 1.91E-02 | 1.00 | 7.45E-12 | 2.27E-09 |
|  | hsa-miR-4299 | 4.87 | 7.59 | 0.15 | -2.73 | 2.33E-04 | 3.55E-02 | 1.00 | 3.88E-11 | 5.91E-09 |
|  | kshv-miR-K12-7-5p | 2.3 | 4.72 | 0.19 | -2.43 | 1.61E-02 | 7.71E-02 | 1.00 | 6.99E-10 | 3.04E-08 |
|  | hsa-miR-1973 | 3.77 | 6.10 | 0.20 | -2.33 | 1.17E-02 | 7.11E-02 | 1.00 | 2.31E-10 | 1.76E-08 |
|  | hsa-miR-6785-5p | 7.74 | 9.92 | 0.22 | -2.18 | 5.69E-03 | 6.15E-02 | 1.00 | 2.21E-10 | 1.76E-08 |
|  | hsa-miR-6894-5p | 2.86 | 5.01 | 0.23 | -2.15 | 8.74E-04 | 5.35E-02 | 1.00 | 2.91E-10 | 1.97E-08 |
|  | hsa-miR-4485 | 8.43 | 10.53 | 0.23 | -2.10 | 9.88E-04 | 5.35E-02 | 1.00 | 3.58E-10 | 2.18E-08 |
|  | hsa-miR-664b-5p | 2.42 | 4.50 | 0.24 | -2.09 | 9.67E-03 | 6.67E-02 | 1.00 | 4.25E-10 | 2.35E-08 |
|  | hbv-miR-B26-5p | 7.63 | 9.60 | 0.25 | -1.97 | 2.92E-04 | 3.55E-02 | 1.00 | 5.44E-10 | 2.55E-08 |
|  | Hsv1-miR-H18 | 2.93 | 4.75 | 0.28 | -1.82 | 6.35E-03 | 6.15E-02 | 1.00 | 3.60E-09 | 1.21E-07 |
|  | hsa-miR-513a-5p | 2.62 | 4.42 | 0.29 | -1.80 | 4.02E-02 | 1.21E-01 | 1.00 | 1.54E-08 | 3.29E-07 |
|  | hsa-miR-4428 | 2.50 | 4.15 | 0.32 | -1.64 | 5.07E-03 | 6.15E-02 | 1.00 | 2.89E-09 | 1.04E-07 |
|  | hsa-miR-4728-5p | 3.94 | 5.58 | 0.32 | -1.64 | 2.18E-03 | 6.15E-02 | 1.00 | 6.81E-09 | 1.81E-07 |
|  | hsa-miR-4787-5p | 7.27 | 8.83 | 0.34 | -1.56 | 1.79E-02 | 8.11E-02 | 1.00 | 1.02E-08 | 2.57E-07 |
|  |  |  |  |  |  |  |  |  |  |  |
| SMAD4-associated up-regulated miRNA | hsa-miR-5585-3p | 6.07 | 2.98 | 8.57 | 3.10 | 9.43E-03 | 6.67E-02 | 0.00 | 6.87E-11 | 8.36E-09 |
|  | hsa-miR-1260a | 8.37 | 5.93 | 5.41 | 2.44 | 4.44E-03 | 6.15E-02 | 0.00 | 1.45E-10 | 1.47E-08 |
|  | hsa-miR-328-5p | 10.21 | 8.04 | 4.48 | 2.17 | 8.49E-03 | 6.54E-02 | 0.00 | 1.19E-09 | 4.85E-08 |
|  | hsa-miR-762 | 9.66 | 7.51 | 4.45 | 2.15 | 1.10E-02 | 6.95E-02 | 0.00 | 5.37E-10 | 2.55E-08 |
|  | hsa-miR-345-3p | 4.87 | 2.86 | 4.04 | 2.02 | 5.74E-03 | 6.15E-02 | 0.00 | 1.63E-08 | 3.29E-07 |
|  | hsa-miR-6090 | 14.20 | 12.31 | 3.71 | 1.89 | 4.87E-02 | 1.29E-01 | 0.00 | 4.65E-08 | 6.02E-07 |
|  | hsa-miR-150-3p | 7.68 | 5.91 | 3.40 | 1.77 | 4.70E-03 | 6.15E-02 | 0.00 | 1.49E-09 | 5.67E-08 |
|  | hsa-miR-6850-5p | 8.81 | 7.05 | 3.38 | 1.76 | 6.46E-02 | 1.46E-01 | 0.00 | 1.37E-07 | 1.33E-06 |
|  | hsv2-miR-H6-5p | 8.67 | 6.95 | 3.29 | 1.72 | 5.73E-02 | 1.35E-01 | 0.00 | 8.56E-08 | 8.83E-07 |
|  | hsa-miR-4454 | 15.31 | 13.60 | 3.26 | 1.71 | 1.95E-01 | 2.94E-01 | 0.00 | 2.24E-04 | 5.83E-04 |
|  | hsa-miR-1260b | 7.53 | 5.91 | 3.07 | 1.62 | 7.21E-03 | 6.46E-02 | 0.00 | 1.78E-08 | 3.29E-07 |
|  | hsa-miR-7977 | 13.55 | 11.94 | 3.06 | 1.61 | 2.64E-02 | 9.73E-02 | 0.00 | 6.82E-09 | 1.81E-07 |
|  | hbv-miR-B2RC | 4.89 | 3.29 | 3.03 | 1.60 | 2.15E-02 | 8.59E-02 | 0.00 | 5.08E-09 | 1.55E-07 |
|  | hsa-miR-3188 | 6.03 | 4.51 | 2.88 | 1.52 | 5.49E-04 | 5.35E-02 | 0.00 | 3.77E-09 | 1.21E-07 |
