# Supplementary material for: PDAC-derived exosomes enrich the microenvironment in MDSCs in a SMAD4-dependent manner through a new calcium related axis
Source: Oncotarget. 2017 Sep 13;8(49):84928–44. doi: 10.18632/oncotarget.20863 (PMC5689584; doi:10.18632/oncotarget.20863)
Supplement: Supplementary file 7 [file oncotarget-08-84928-s007.docx]

**Supplementary Table 6.** Antibodies and related isotypes. FITC: fluorescein isothiocyanate, PE: phycoerythrin, ECD: phycoerythrincovalently linked to Texas red, PC5: phycoerythrincovalently linked to cyanine 5, PC7: phycoerythrin covalently linked to cyanine 7. All antibodies are produced by Beckman Coulter, Miami, FL, USA except * BD Biosciences, San Josè, CA, USA; † Invitrogen, Carlsbad, CA, USA.

| **Antibody** | **Fluorochrome** | **Isotype** |
| --- | --- | --- |
| Anti-CD3 | ECD | IgG1 |
| Anti-CD4* | FITC | IgG1* |
| Anti-CD11b | PE | IgG1 |
| Anti-CD14 | PC7 | IgG2a |
| Anti-CD25* | PE | IgG1* |
| Anti-CD45 | ECD | IgG1 |
| Anti-CD45^†^ | PC5 | IgG1^†^ |
| Anti-HLA-DR | PC5 | IgG1 |
| Anti-CD16 | FITC | IgG1 |
| Anti-CD80 | FITC | IgG1 |
| Anti-CD86 | PE | IgG2b |
| Anti-CD45-FITC/CD4-PE/  CD8-ECD/CD3-PC5  (Cyto-Stat tetraCHROME) | | IgG2b  IgG1 |
